# The Epstein-Barr Virus BRRF1 Gene Is Dispensable for Viral Replication in HEK293 cells and Transformation

**DOI:** 10.1038/s41598-017-06413-7

**Published:** 2017-07-20

**Authors:** Masahiro Yoshida, Takahiro Watanabe, Yohei Narita, Yoshitaka Sato, Fumi Goshima, Hiroshi Kimura, Takayuki Murata

**Affiliations:** Department of Virology, Nagoya University Graduate School of Medicine, 65 Tsurumai-cho, Showa-ku, Nagoya 466-8550 Japan

## Abstract

The Epstein-Barr virus (EBV) is a gamma-herpesvirus associated with several malignancies. It establishes a latent infection in B lymphocytes and is occasionally reactivated to enter the lytic cycle. Here we examined the role of the EBV gene BRRF1, which is expressed in the lytic state. We first confirmed, using a DNA polymerase inhibitor, that the BRRF1 gene is expressed with early kinetics. A BRRF1-deficient recombinant virus was constructed using a bacterial artificial chromosome system. No obvious differences were observed between the wild-type, BRRF1-deficient mutant and the revertant virus in HEK293 cells in terms of viral lytic protein expression, viral DNA synthesis, progeny production, pre-latent abortive lytic gene expression and transformation of primary B cells. However, reporter assays indicated that BRRF1 may activate transcription in promoter- and cell type-dependent manners. Taken together, BRRF1 is dispensable for viral replication in HEK293 cells and transformation of B cells, but it may have effects on transcription.

## Introduction

The Epstein-Barr virus (EBV) is a ubiquitous human gamma-herpesvirus. Over 90% of the world’s population is latently infected with EBV. After primary infection, EBV establishes a latent infection in B lymphocytes. Primary infection with EBV causes infectious mononucleosis, and latent infection is associated with several human malignancies including malignant lymphoma, gastric carcinoma and nasopharyngeal carcinoma^1, 2^.

The EBV has two alternative life cycles: latent and lytic^1, 3^. During the latent state, an episome of the EBV DNA exists in the nucleus and expresses only a few latent proteins. Induction of the lytic cycle from the latent state is called reactivation^4^. The reactivation of EBV is induced in cell culture by chemical or biological agents, including histone deacetylase inhibitors, 12-O-tetradecanoylphorbol-13-acetate (TPA), and calcium ionophores^1, 5, 6^. The exogenous expression of the immediate-early (IE) gene BZLF1^7^ (Zta, Z, ZEBRA, EB1) or BRLF1^8^ (Rta, R) can also induce reactivation. IE genes induce expression of early (E) genes, such as BMRF1, BALF2 and BHRF1. These E gene products induce replication of the EBV genome as a concatemer. After replication, late (L) genes^9^, which encode viral structural proteins, such as major capsid protein (MCP), gp350 and gB, are expressed. The EBV DNA is incorporated into assembled icosahedral capsid structures. The nucleocapsids bud into the nuclear membrane and are then enveloped with tegument proteins and glycoproteins to form progeny viral particles.

Soon after primary infection, EBV enters a transient lytic state^10^ called the pre-latent abortive lytic cycle. In this cycle, latent genes and some lytic genes are expressed. This transient lytic state is silenced by chromatinization, and EBV establishes a latent infection.

*In vitro*, human B lymphocytes are transformed into lymphoblastoid cell lines (LCLs), which grow continuously, after EBV infection. This process is called immortalization or transformation.

Although latent genes have been investigated extensively, some lytic genes, such as BRRF1 (also designated Na) have not been well characterized. The BRRF1 gene, encoding a 34 kDa protein, is located between the BRLF1 and BRRF2 coding regions^11^. The promoter driving BRRF1 expression is located within the coding sequence of BRLF1 and is activated by BZLF1. BRRF1 has been reported to localize to the nucleus in HeLa cells^11^ or to the nucleus and cytoplasm in Hone-Akata cells^12^.

Using a BRLF1 and BRRF1 double-knockout mutant virus, a previous study reported that BRRF1 enhanced BRLF1-induced reactivation in HEK293 cells and a gastric carcinoma cell line, but not in lymphoblastoid or Burkitt cell lines^10^. A reporter assay performed in HeLa cells revealed that BRRF1 activates the BZLF1 promoter through a cAMP response element (CRE) motif by enhancing the transcriptional function of c-Jun, which in combination with c-Fos forms activation protein 1 (AP-1)^13^. Knockdown of endogenous BRRF1 inhibited lytic reactivation induced by chemical substances in nasopharyngeal carcinoma cell lines^12^. BRRF1 was also reported to induce lytic protein expression by itself^12^. Although BRRF1 did not affect p53 function in the reporter assay, BRRF1 required p53 to induce reactivation^12^. However, the mechanism of BRRF1 action has not been fully elucidated using BRRF1 single knockout viruses.

To determine the role of the BRRF1 gene, BRRF1-knockout EBV strains were engineered and compared with wild-type and revertant viruses. The BRRF1- deficient mutant showed no significant differences in phenotype compared with the wild-type virus. However, our reporter assays showed that promoters containing response elements of p53, AP-1, CREB and NF-κB were activated by BRRF1 to some extent. Therefore, BRRF1 is dispensable for viral replication, at least in HEK293 cells, and in the transformation of primary B cells, but it may have effects on transcription.

## Results

### BRRF1 is expressed with early kinetics

**Figure 1:**
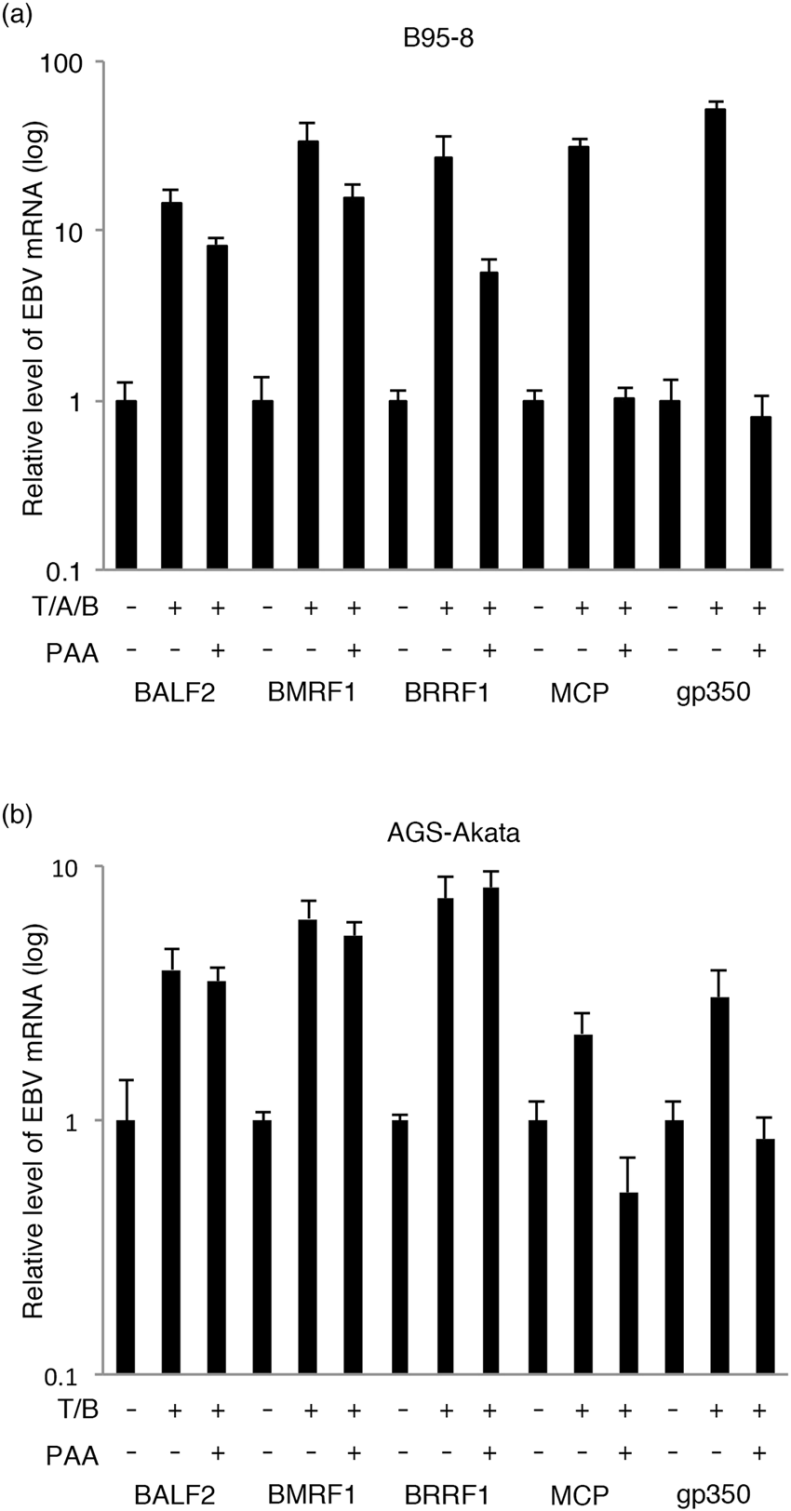
BRRF1 is expressed with early kinetics. (**a**) B95-8 cells were lytically induced by TPA, A23187 and butyrate (T/A/B) with or without PAA^14^. (**b**) AGS-Akata cells were lytically induced by TPA and butyrate (T/B) with or without PAA. Cellular RNA was collected after 48 hours, and qRT-PCR was performed using BALF2, BMRF1, BRRF1, MCP, gp350 and GAPDH primers. Relative mRNA levels for each gene are shown after normalization to GAPDH mRNA levels. Each bar represents the mean and standard deviation of three independent experiments.

To examine the expression kinetics of the BRRF1 gene, B95-8 cells were lytically induced by TPA, A23187 (a calcium ionophore) and sodium butyrate (T/A/B) with or without phosphonoacetic acid (PAA), a DNA polymerase inhibitor, for 48 hours (Fig. 1a) as described previously^14^. Total RNA was subjected to quantitative reverse transcription PCR (qRT-PCR). mRNA expression of the E genes (BALF2 and BMRF1) was induced more than 10-fold by T/A/B treatment and was only marginally inhibited by the addition of PAA (Fig. 1a). Additionally, the L genes (MCP and gp350) were induced by lytic induction but were almost totally inhibited by PAA (Fig. 1a). The BRRF1 mRNA levels induced by lytic stimulation were only partly decreased by PAA in B95-8 cells (Fig. 1a).

We next evaluated AGS-Akata cells (Fig. 1b). While induction of L genes (MCP and gp350) by TPA and sodium butyrate (T/B) was suppressed by PAA, induction of E genes (BALF2 and BMRF1) and BRRF1 was not influenced by PAA treatment (Fig. 1b). These results indicate that the BRRF1 gene is expressed with early but not late kinetics as suggested previously^13, 15^.

### Construction of BRRF1 knockout virus

**Figure 2:**
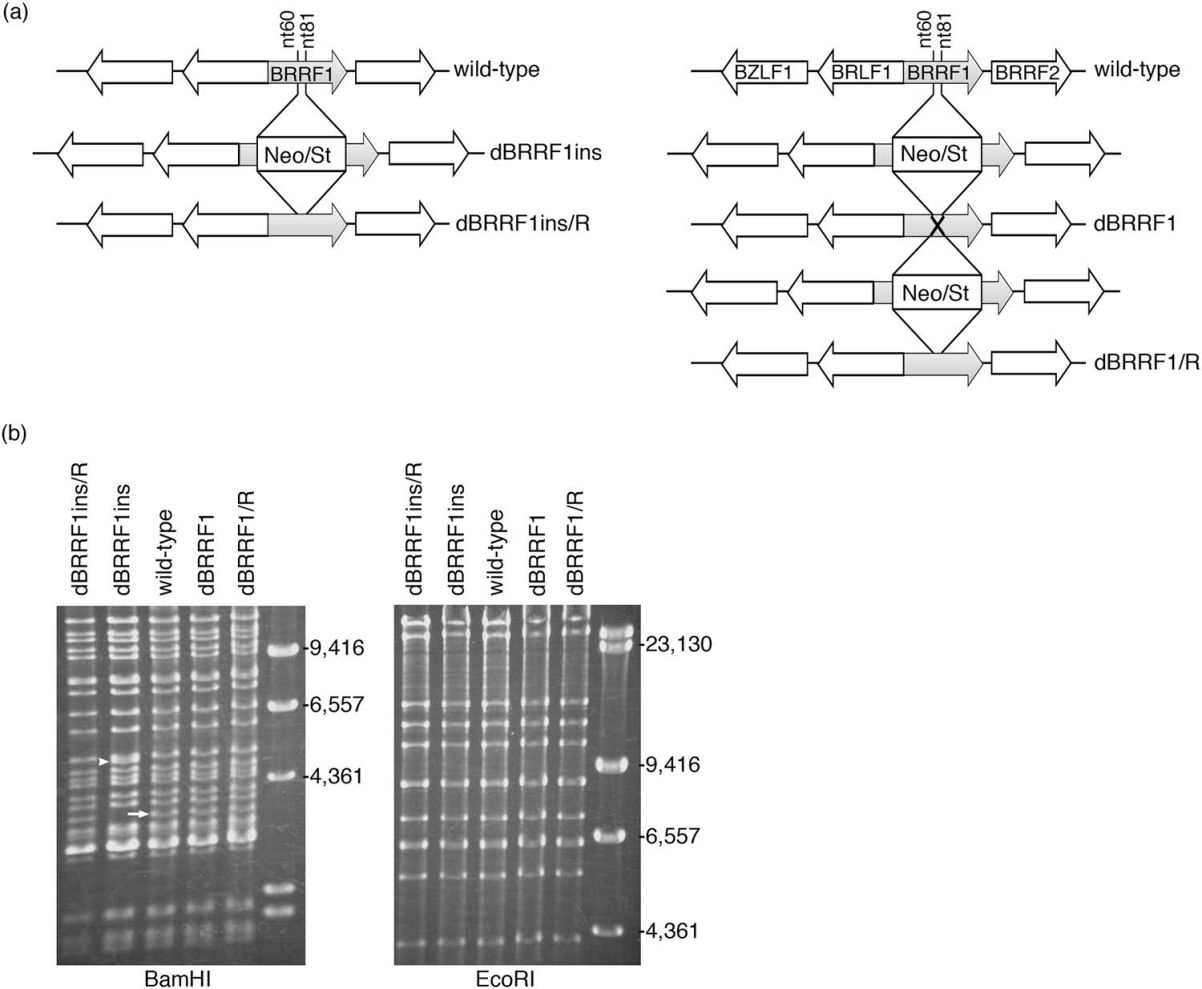
Construction of BRRF1-knockout EBV. (**a**) BRRF1 knockout EBV and the revertant were constructed using the BAC system in *Escherichia coli* ^22, 25^. The Neo/St cassette, which contains neomycin resistance and streptomycin sensitivity genes, was inserted between nts 60 and 81 of the BRRF1 gene (dBRRF1ins). Then, the cassette was replaced with the original BRRF1 sequence (dBRRF1ins/R) or with a BRRF1 sequence containing two stop codons, nts 72 T > A and 76 G > T (dBRRF1). The Neo/St cassette was again inserted and replaced with the original BRRF1 sequence (dBRRF1/R). (**b**) EBV BAC DNAs were digested with BamHI or EcoRI and analysed by electrophoresis. The BamHI-R fragment of EBV is indicated by a white arrow and an arrowhead. Full-length gels are presented in Supplementary Figure 8.

To analyse the function of BRRF1, we next constructed two types of BRRF1 knockout viruses (cassette insertion and nonsense mutants) and their corresponding revertants using a bacterial artificial chromosome (BAC) system (Fig. 2a). The insertion mutant virus (dBRRF1ins) was made by simply inserting the Neo/St cassette, which contains neomycin resistance and streptomycin sensitivity genes, between nucleotides (nt) 60 and 81 of the BRRF1 gene (1–933 nt in total) (Fig. 2a left panel). Then, the cassette was replaced with the original BRRF1 sequence to prepare the repaired strain (dBRRF1ins/R). For the nonsense mutant strain, the Neo/St cassette was inserted as described above and then replaced with a BRRF1 sequence containing two stop codons, nts 72 T > A and 76 G > T (dBRRF1) (Fig. 2a right panel). The Neo/St cassette was again inserted and replaced with the wild-type BRRF1 sequence to prepare the revertant virus genome (dBRRF1/R).

These recombinant EBV BAC DNAs were digested with BamHI or EcoRI and analysed by electrophoresis (Fig. 2b). The BamHI-R fragment of EBV was present in wild-type, dBRRF1, dBRRF1/R and dBRRF1ins/R viruses (white arrow), but the band corresponding to the BamHI-R fragment from the dBRRF1ins virus (white arrowhead) migrated slowly. The band pattern after EcoRI digestion was almost identical, because the Neo/St cassette was inserted into the second largest fragment; therefore, the two fragments were indistinguishable on the agarose gel. The expected sequence of the EBV BAC DNA from each recombinant virus was confirmed.

Recombinant EBV BAC DNA was transfected into HEK293 cells by lipofection, and hygromycin-resistant cells were cloned for further analysis. After establishment of HEK293 cell lines containing recombinant EBV BAC, the BRRF1 gene in the cell lines was again sequenced for further validation. We confirmed that the cell lines harboring the BRRF1-knockout viruses do not carry the wild-type sequences, and that cell lines harboring the wild-type and revertant mutants have intact BRRF1 sequences.

### BRRF1 knockout or knockdown did not markedly affect lytic replication by BZLF1

**Figure 3:**
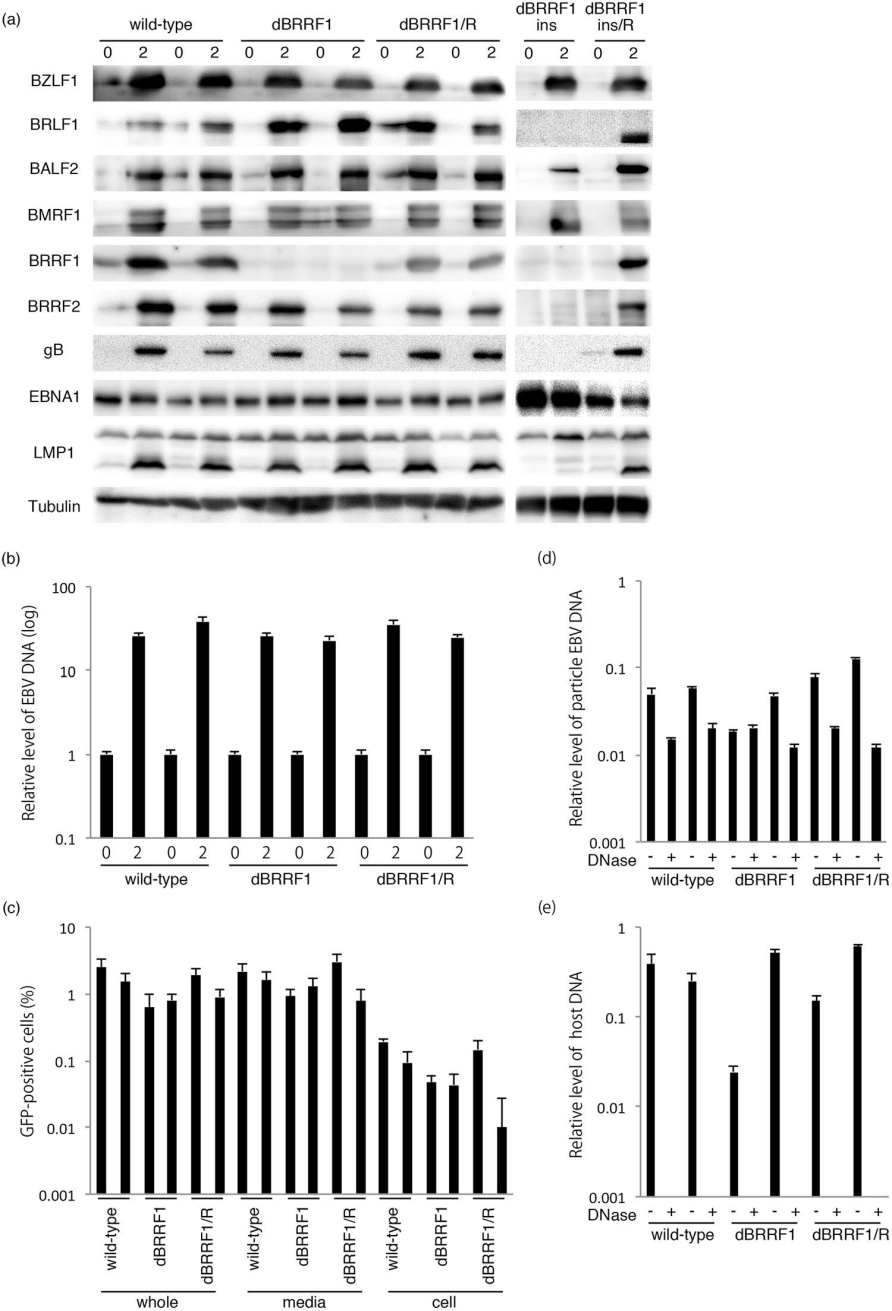
Lytic protein expression, EBV DNA synthesis, progeny production and extracellular virion DNA in wild-type, dBRRF1 and dBRRF1/R viruses after forced expression of BZLF1. (**a**) HEK293 cells harboring the EBV BAC were transfected with the BZLF1 expression vector. Cells were collected on days 0 and 2 and subjected to immunoblotting. The original blots are presented in Supplementary Figures 9–12. (**b**) Cells were collected on days 0 and 2 and subjected to qPCR. Each bar represents the mean and standard deviation of three independent experiments. (**c**) Viruses were collected from the media (media), cells (cell) or both (whole) on day 3. After a freeze-thaw process and centrifugation, supernatants were cultured with Akata(-) cells. GFP-positive cells, representing the progeny virus titres, were counted using FACS. Each bar represents the mean and standard deviation of three independent experiments. Representative FACS results are presented in Supplementary Figure 13. (**d**,**e**) A portion of the medium sample from (**c**) was digested with Turbo DNase I. After DNA purification, EBV DNA (**d**) and host genome DNA levels (**e**) were analysed by qPCR. Each bar represents the mean and standard deviation of three independent qPCR experiments.

To characterize the recombinant viruses, lytic viral protein expression was determined by immunoblotting (Fig. 3a). Two typical clones harboring each EBV-BAC (wild-type, dBRRF1 and dBRRF1/R) were chosen for analysis. After lytic induction by BZLF1, cells were collected on days 0 and 2. BRRF1 protein was absent in dBRRF1 and dBRRF1ins and present in wild-type, dBRRF1/R and dBRRF1ins/R, as expected (Fig. 3a). However, BRLF1 and BRRF2 proteins were also absent in the cassette insertion knockout mutant (dBRRF1ins), indicating that insertion of the cassette in the BRRF1 gene hindered expression of neighbouring genes (Fig. 3a). It was also assumed that restricted expression of BRLF1 in the insertion mutant (dBRRF1ins) caused inhibition of gB and the lytic LMP1 (lower band in the LMP1 blot detected after induction in the other samples) (Fig. 3a). Therefore, this insertion mutant and the revertant were not used for further experiments. On the other hand, BRLF1 and BRRF2 proteins were not reduced in the BRRF1-deficient viruses containing a stop codon (dBRRF1). The expression of BZLF1, BALF2, BMRF1, gB, EBNA1 and LMP1 genes were comparable 2 days after induction (Fig. 3a). This indicates that knockout of BRRF1 does not affect viral protein expression after induction by BZLF1.

Next, we analysed the levels of EBV DNA synthesis in HEK293 cells harboring recombinant viruses. After lytic induction by BZLF1, cells were collected on days 0 and 2 for DNA analysis. EBV DNA levels of the dBRRF1 virus were comparable to those of wild-type and dBRRF1/R viruses, according to qPCR analysis (Fig. 3b). This indicates that knockout of BRRF1 does not affect EBV DNA replication after induction by BZLF1.

To determine the amount of progeny virus production, HEK293 cells harboring each EBV-BAC clone were lytically induced by BZLF1 expression. Cell-associated and cell-free viruses (whole), cell-free viruses (media) or cell-associated viruses (cell) were collected 3 days after induction. After a freeze-thaw process, cell debris was cleared by centrifugation. Then the samples were co-cultured with EBV-negative Akata cells (Akata(-)). Since the recombinant EBV expresses GFP protein, GFP-positive cells were counted using FACS to determine progeny virus titres. Progeny viral levels in the dBRRF1 virus were not significantly different from those in the wild-type or dBRRF1/R viruses (Fig. 3c). We also analysed the amount of progeny production 1, 3 or 5 days after induction by BZLF1 in HEK293 cells harboring wild-type or dBRRF1 recombinant viruses. The peak of progeny production was on day 3. The amount and time course of progeny production in the dBRRF1 virus were not significantly different from those in the wild-type virus (Supplementary Figure 1).

To further extend this result, we determined the levels of EBV DNA in the virus particles in media. A portion of the media used for the progeny titration (Fig. 3c) was treated with Turbo DNase I to eliminate naked viral DNA. After purification, EBV DNA levels were analysed by qPCR (Fig. 3d). Extracellular particle DNA levels of the dBRRF1 virus were comparable to those of the wild-type or dBRRF1/R viruses (Fig. 3d). As a control (Fig. 3e), we also examined the level of host 18S DNA in the same samples used in Fig. 3d. DNase treatment successfully eliminated the naked host DNA to <0.01 (Fig. 3e). These results indicate that BRRF1 disruption does not significantly affect the production of progeny virus or extracellular production of EBV after induction by BZLF1.

To confirm these results obtained by using dBRRF1 virus, we also analysed the lytic protein expression, EBV DNA synthesis and progeny production in HEK293 cells harboring wild-type viruses after transfection of BZLF1 expression vector and control siRNA or siRNAs against BRRF1. The knockdown of BRRF1 did not affect viral protein expression, EBV DNA replication and production of progeny virus (Supplementary Figure 2).

### BRRF1 knockout did not markedly affect lytic replication by BRLF1

**Figure 4:**
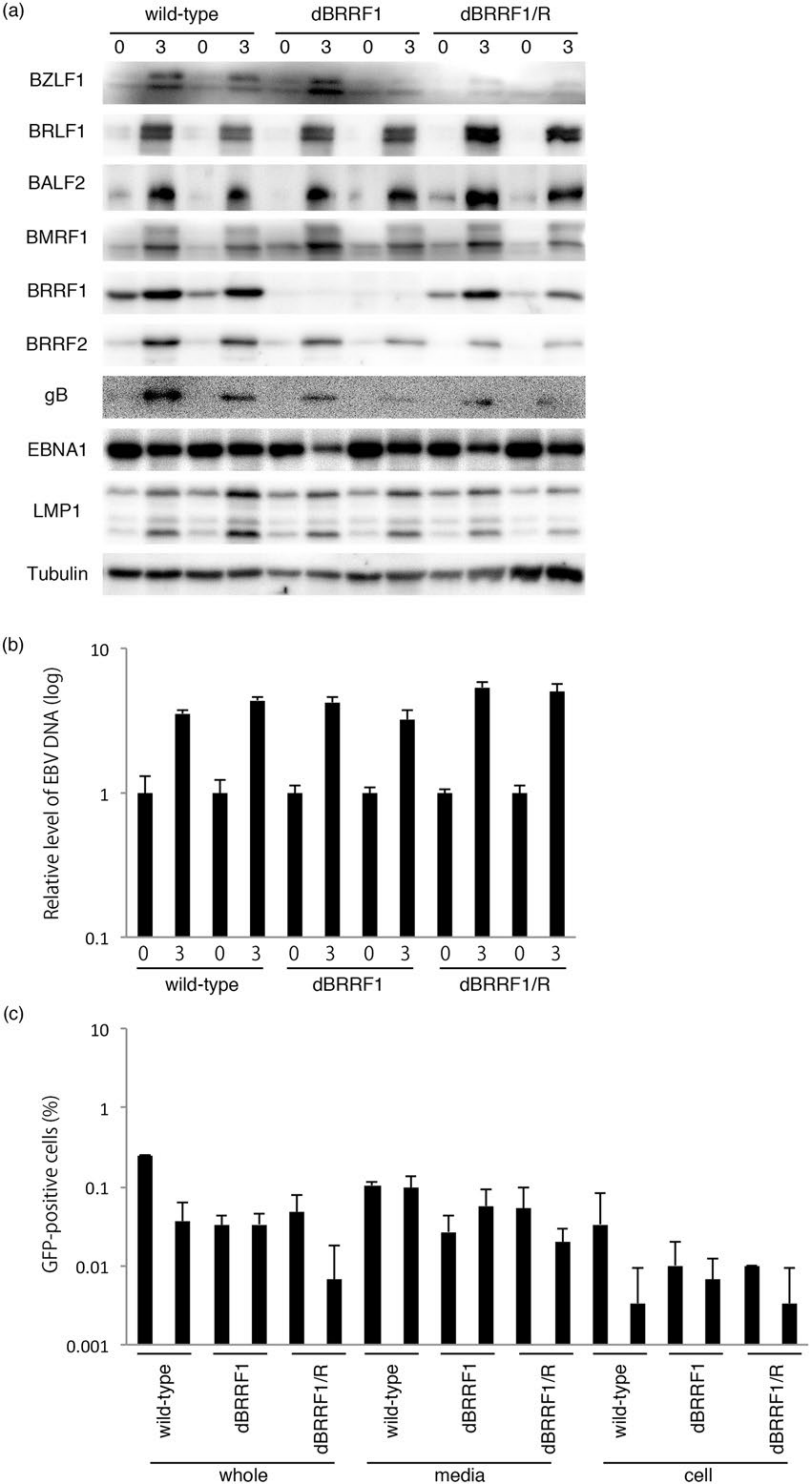
Lytic protein expression, EBV DNA synthesis and progeny production in wild-type, dBRRF1 and dBRRF1/R viruses after forced expression of BRLF1. (**a**) HEK293 cells harboring the EBV BAC were transfected with the BRLF1 expression vector. Cells were collected on days 0 and 3 and subjected to immunoblotting. The original blots are presented in Supplementary Figures 14 and 15. (**b**) DNA from cells collected on days 0 and 3 was used for qPCR. Each bar represents the mean and standard deviation of three independent experiments. (**c**) Viruses were collected from the media (media), cells (cell) or both (whole) on day 4. After a freeze-thaw process and centrifugation, the supernatants were cultured with Akata(-) cells. GFP-positive cells, representing progeny virus titres, were counted using FACS. Each bar represents the mean and standard deviation of three independent experiments.

In the previous section, we showed that induction of the EBV lytic replication cycle by BZLF1 was not significantly influenced by disruption of the BRRF1 gene (Fig. 3). Since a previous report implied, using a BRRF1/BRLF1 double knockout virus, that BRRF1 might enhance the EBV lytic replication induced by BRLF1^13^, we assessed whether our point-mutated virus might be affected upon BRLF1 induction. Two typical clones of each EBV BAC (wild-type, dBRRF1 and dBRRF1/R) were transfected with the BRLF1 expression vector, and cells were collected on days 0 and 3 for protein analysis. As expected, the BRRF1 protein was absent in dBRRF1 but present in wild-type and dBRRF1/R viruses (Fig. 4a). The expression levels of BZLF1, BRLF1, BALF2, BMRF1, BRRF2, gB, EBNA1 and LMP1 genes were comparable 3 days after induction (Fig. 4a).

We also analysed the levels of EBV DNA synthesis. After lytic induction by BRLF1, cells were collected on days 0 and 3 for DNA analysis. qPCR analysis showed that EBV DNA levels of the dBRRF1 virus were comparable to those of the wild-type or dBRRF1/R viruses (Fig. 4b).

The level of progeny was not lower for the dBRRF1 virus compared with the wild-type or dBRRF1/R virus (Fig. 4c).

For the experiments shown in Fig. 4, we collected protein and viral DNA samples after 3 days and progeny samples after 4 days, which were longer incubation times than those specified in Fig. 3. We used longer incubation times because induction of the lytic cycle by BRLF1 was much weaker compared with that by BZLF1.

These results indicate that disruption of BRRF1 does not significantly affect induction of lytic replication in HEK293 cells by BRLF1.

### BRRF1 knockout did not affect pre-latent abortive lytic gene expression or transformation efficacy

**Figure 5:**
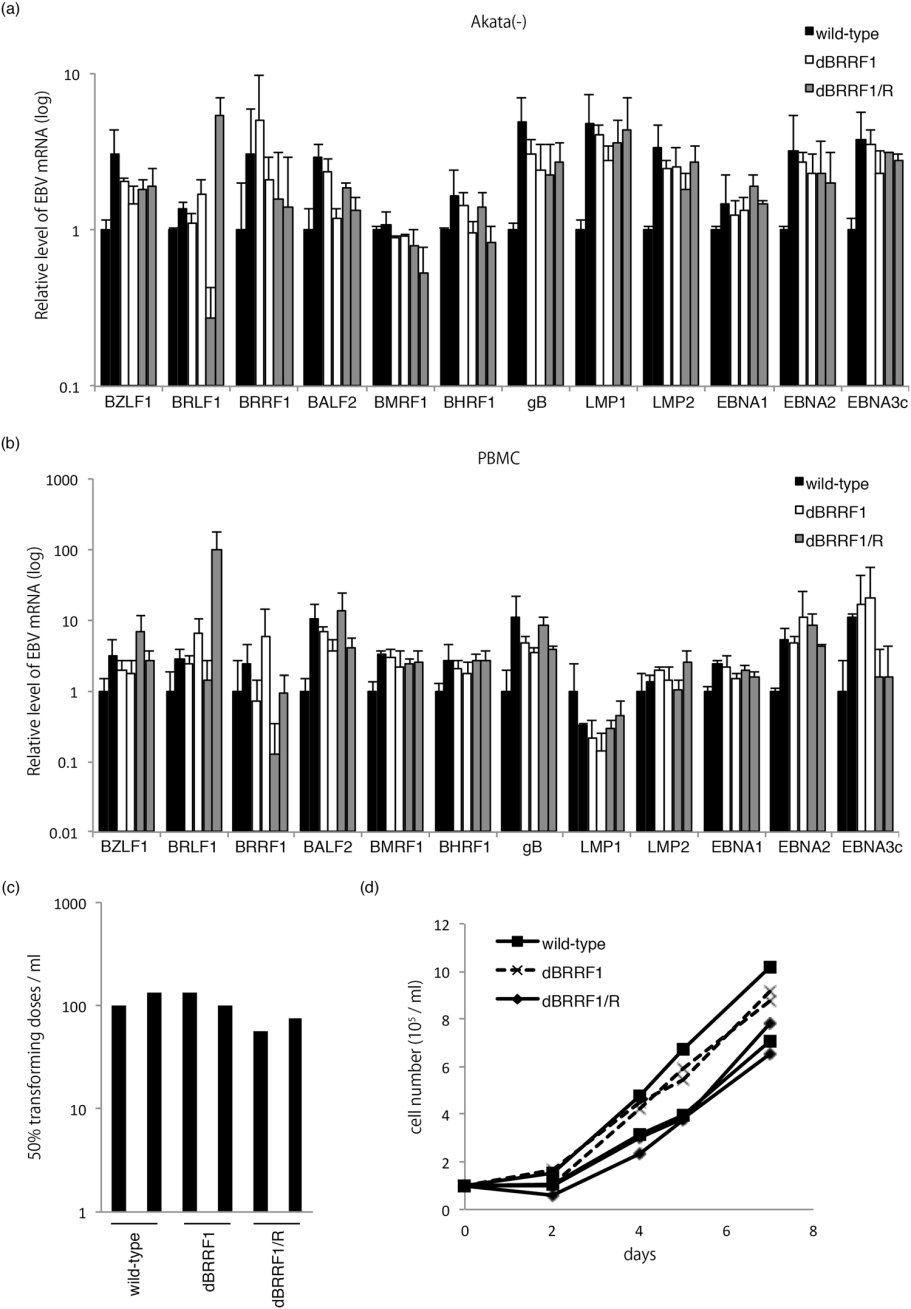
Pre-latent abortive lytic gene expression and transformation properties of wild-type, dBRRF1 and dBRRF1/R viruses. To prepare virus stock solution, HEK293 cells harboring the EBV BAC were transfected with the BZLF1 expression vector. Virus stock solution was collected from two independent HEK293 EBV BAC clones for each strain. Supernatants were collected on day 3, followed by titration. The virus solutions of equivalent titres were used to infect Akata(-) cells (**a**) or PBMCs (**b**). Total RNA was collected after 48 hours, and qRT-PCR was performed. Relative mRNA levels for each gene are shown after normalization to GAPDH mRNA levels. Each bar represents the mean and standard deviation of three independent experiments. (**c**) Transformation efficacy of the indicated viruses. PBMCs from a healthy donor were infected with 10-fold serial dilutions of the normalized virus stock. Transforming titres were determined 25 days later. The bar represents the transformation units in the virus stock solution prepared from two independent clones of EBV-harboring HEK293 cells for each recombinant EBV. (**d**) Growth curves for LCLs. LCLs prepared in (**c**) were seeded at 1 × 10^5^/ml and counted after 2, 4, 5 and 7 days.

Upon infection and before establishment of solid latency, EBV-infected cells enter the so-called pre-latent abortive lytic state, in which a number of lytic genes are expressed, essentially in the absence of viral DNA replication. We next evaluated if lytic gene expression in the pre-latent abortive lytic cycle could be affected by disruption of the BRRF1 gene. Titres of virus stock solutions obtained from HEK293 cells harboring EBV BAC clones after BZLF1 expression were determined, and Akata(-) cells or peripheral blood mononuclear cells (PBMCs) were infected with viruses (Fig. 5a,b) after normalization. Total RNA was collected and subjected to qRT-PCR 2 days after infection. We confirmed that the transcription levels of IE genes (BZLF1 and BRLF1), E genes (BRRF1, BALF2, BMRF1 and BHRF1), an L gene (gB) and latent genes (LMP1, LMP2, EBNA1, EBNA2 and EBNA3c) from the dBRRF1 virus were not significantly different from those of the wild-type and dBRRF1/R viruses (Fig. 5a,b). These results indicate that BRRF1 does not significantly affect viral RNA transcription in the pre-latent abortive lytic cycle.

We next examined the transforming efficacy of the recombinant viruses. PBMCs from a healthy donor were infected with normalized virus stock solutions obtained from HEK293 cells after BZLF1 expression, and the transformation unit was calculated after 25 days. The transformation efficacy of the dBRRF1 virus was almost equal to those of the wild-type and dBRRF1/R viruses (Fig. 5c). We then analysed growth of the LCLs after expansion. LCLs prepared as described in Fig. 5c were seeded at 1 × 10^5^/ml and counted after 2, 4, 5 and 7 days. The growth curves of LCLs transformed with the dBRRF1 virus were comparable to those of LCLs transformed with the wild-type and dBRRF1/R viruses (Fig. 5d). These results indicate that BRRF1 does not significantly affect EBV-induced B cell transformation or LCL growth.

### Effect of BRRF1 on BZLF1 promoter activity in reporter assays

**Figure 6:**
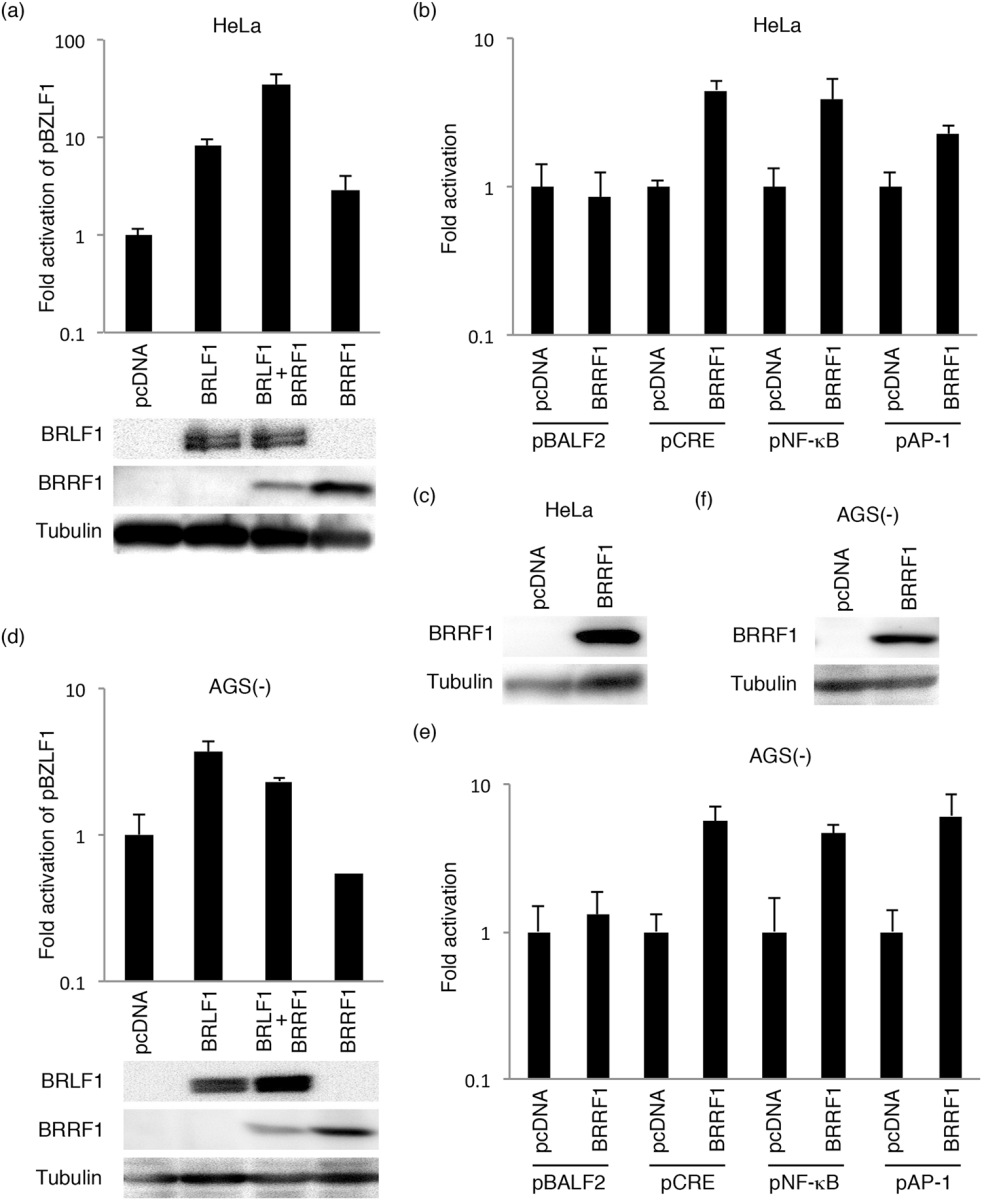
Evaluation of promoter activity. The indicated expression vectors were transfected by lipofection into HeLa (**a**,**b**,**c**) or AGS(-) (**d**,**e**,**f**) cells, along with pBZLF1-FL (**a**,**d**) or each reporter plasmid (pBALF2-FL, pCRE-FL, pNF-κB-FL and pAP1-FL) (**b**,**e**) and null-RL. Cells were collected after 24 hours and subjected to luciferase assays. FL activity was normalized to RL, and the values for the cells transfected with the empty vector (pcDNA3) were set to 1. (**a**,**c**,**d**,**f**) Cell lysates were subjected to immunoblotting, and a representative result is presented. Original blots are presented in Supplementary Figures 16–19.

To determine if BRRF1 can activate the BZLF1 promoter, as reported previously^13^, we performed luciferase assays in HeLa (Fig. 6a) and EBV-negative AGS cells (AGS(-)) (Fig. 6d), using reporter constructs in which the BZLF1 promoter was positioned upstream of the firefly luciferase gene (pBZLF1-FL). Each cell line was transfected with the indicated expression vector, pBZLF1-FL and control renilla vector. BRLF1 expression increased BZLF1 promoter activity in both HeLa and AGS, but the effect of BRRF1 was cell type-dependent. In HeLa cells, BRRF1 expression caused mild activation of the promoter, and co-expression of BRLF1 and BRRF1 caused further activation (Fig. 6a), as reported previously^13^. However, in AGS cells, BRRF1 did not augment promoter activity regardless of BRLF1 expression (Fig. 6d). Therefore, BRRF1 may be able to activate the BZLF1 promoter at least in certain cell types.

### BRRF1 activated promoters containing CREB, NF-κB and AP-1 response elements

To determine if BRRF1 activates other promoters, we performed further analyses using other reporter plasmids containing the viral BALF2 promoter or promoters with response elements of certain transcription factors (CREB, NF-κB and AP-1). HeLa and AGS(-) cells were transfected as indicated (Fig. 6b,e). Although BRRF1 did not significantly enhance activity of the BALF2 promoter, CRE-, NF-κB- and AP-1-dependent promoters were activated by BRRF1 to some extent in HeLa and AGS cells (Fig. 6b,c,e,f). Therefore, BRRF1 may contribute to transcriptional activation from certain promoters.

### BRRF1 activated p53 response element-dependent transcription

**Figure 7:**
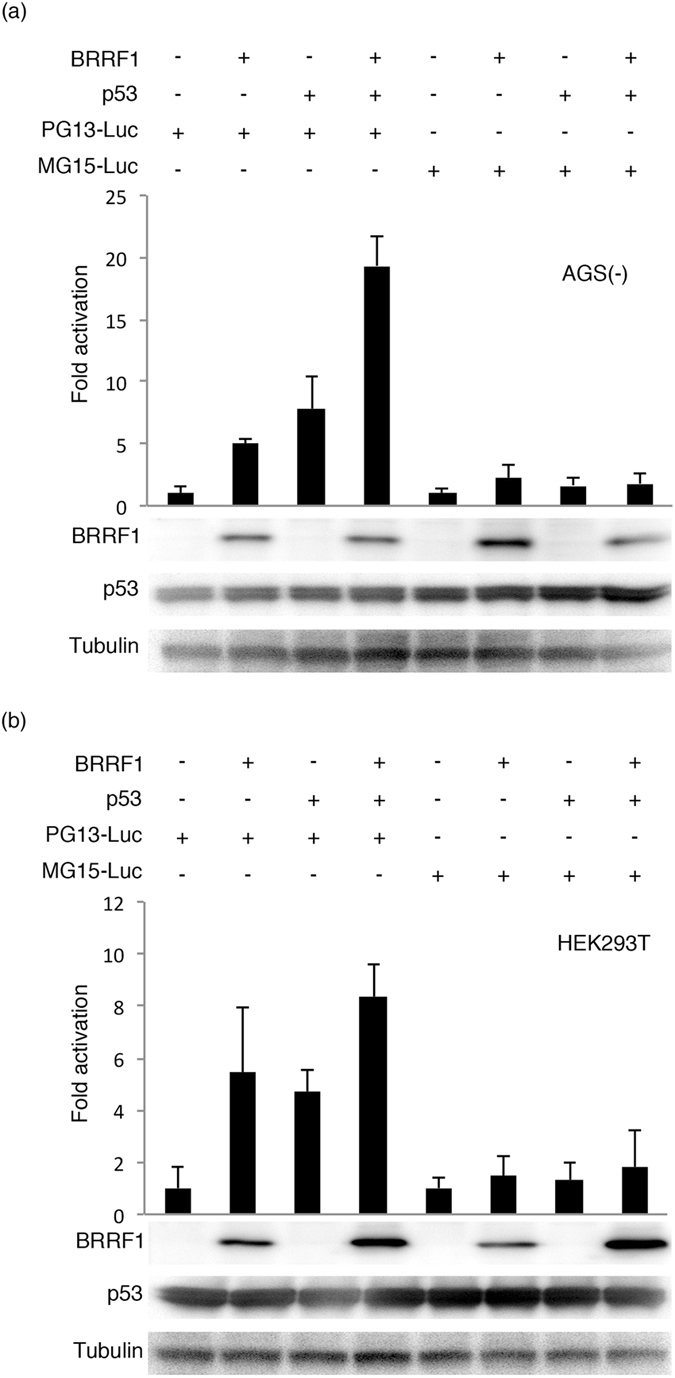
Evaluation of p53-dependent transcription activity. Expression vectors were transfected into AGS(-) (**a**) or HEK293T (**b**) cells along with PG13-Luc, containing 13 wild-type p53 response elements, or MG15-Luc, containing 15 mutated p53 response elements, and null-RL by lipofection. Cells were collected after 24 hours and subjected to luciferase assays. FL activity was normalized to RL, and the values for the cells transfected with the empty vector (pcDNA3) were set to 1. Cell lysates were subjected to immunoblotting, and a representative result is presented below the graph. The original blots are presented in Supplementary Figures 20 and 21.

To determine if BRRF1 activates p53 response elements, we performed luciferase assays in AGS(-) (Fig. 7a) and HEK293T cells (Fig. 7b) using PG13-Luc, which contains 13 wild-type p53 response elements upstream of the FL gene, and MG15-Luc, which contains 15 mutated p53 response elements upstream of the FL gene. BRRF1 expression increased PG13-Luc promoter activity, as well as p53-induced activation of the PG13-Luc promoter in AGS(-) (Fig. 7a) and HEK293T cells (Fig. 7b). This indicates that BRRF1 also has the ability to activate transcription from promoters containing p53 response elements. It must be noted that BRRF1 increased transcription from the p53-dependent promoter in the reporter assays, whereas a previous report^12^ did not observe this and found that BRRF1 somehow induced the EBV lytic cycle in a p53-dependent manner.

### BRRF1 induced expression of a subset of genes but did not enhance EBV DNA replication

**Figure 8:**
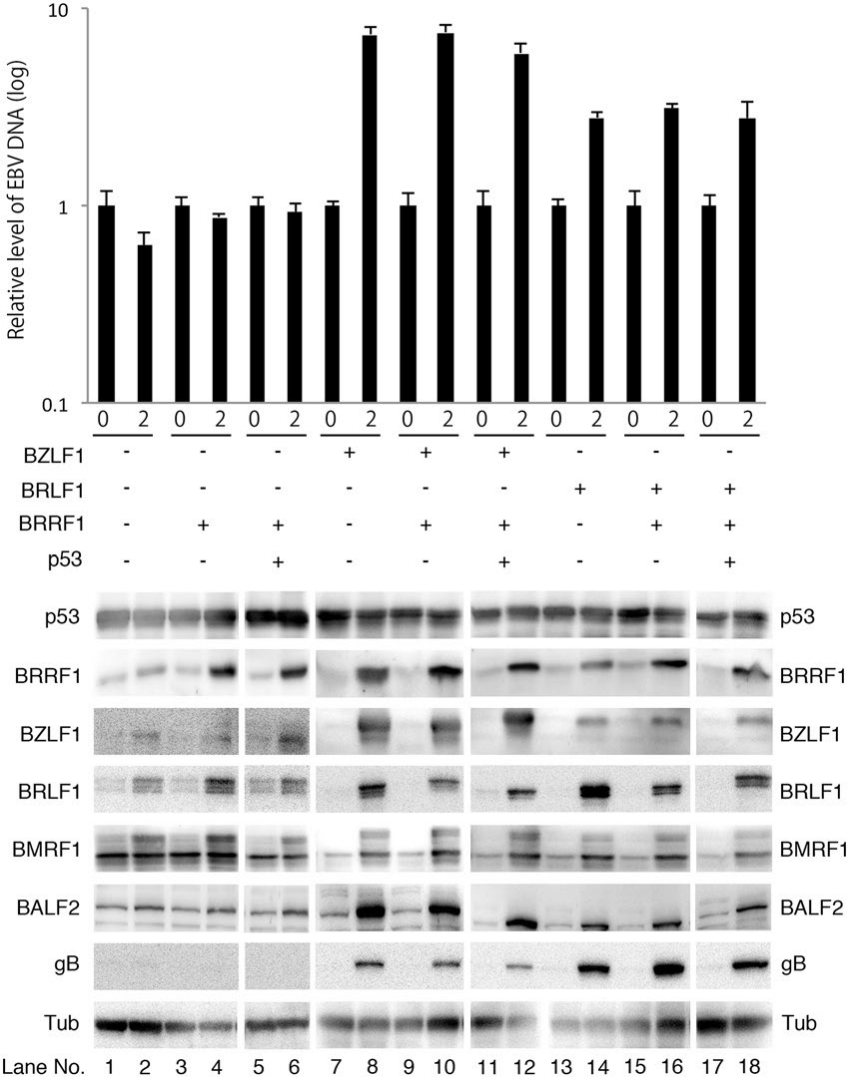
Lytic protein expression and EBV DNA synthesis in HEK293 cells harboring EBV after forced expression of BRRF1 and p53 with/without BRLF1 or BZLF1. HEK293 cells harboring the wild-type EBV BAC were transfected with the indicated expression vector. Cells were collected on days 0 and 2 (or day 3 for the BRLF1 experiment) and subjected to immunoblotting and qPCR. The original blots are presented in Supplementary Figures 22–24. EBV DNA levels were analysed by qPCR. Each bar represents the mean and standard deviation of three independent experiments.

To determine if BRRF1 and/or p53 have the ability to promote lytic infection as suggested previously^12^, we transfected BRRF1 and/or p53 expression vectors with/without BRLF1 or BZLF1 expression vectors into HEK293 cells harboring the wild-type EBV BAC (Fig. 8). Cell samples were collected on days 0 and 2 (or day 3 for the BRLF1 transfection because the induction by BRLF1 was weak) for protein and DNA analysis.

After single transfection of BRRF1, the expression of BZLF1, BRLF1 and BMRF1 was increased to some extent, but the expression of BALF2 and gB was not induced significantly (Fig. 8 lane 4). Forced expression of p53 with BRRF1 did not further increase expression of viral proteins (Fig. 8 lane 6).

We carried out a similar transfection experiment with the addition of BZLF1 (lanes 7–12) and BRLF1 (lanes 13–18). BZLF1 or BRLF1 alone efficiently induced lytic gene expression, but addition of BRRF1 and/or p53 did not further enhance gene expression (Fig. 8).

We also analysed the levels of EBV DNA synthesis (Fig. 8 upper panel). The levels of EBV DNA were not increased after transfection of BRRF1 and/or p53 expression vectors, while efficient viral DNA replication was observed after BZLF1 or BRLF1 transfection (Fig. 8 upper panel).

These results indicate that BRRF1 and/or p53 had the potential to increase the expression of a subset of IE or E genes but could not induce full reactivation. It is possible that, since lytic induction by BZLF1 or BRLF1 was very strong, an increase in lytic cycle induction was not clearly observed with the additional expression of BRRF1 and/or p53.

## Discussion

In this study, we analysed the role of the EBV BRRF1 gene. We first examined the expression kinetics of BRRF1. It was previously reported that the BRRF1 gene is expressed with early kinetics^11, 13^. Our results also support the report that BRRF1 is expressed with early but not late kinetics (Fig. 1), although our data still cannot exclude the possibility that BRRF1 might be an IE gene.

Next, we constructed a BRRF1-deficient recombinant virus using a BAC system (Fig. 2a,b). No obvious differences were observed between wild-type and mutant viruses in terms of viral protein expression (Figs 3a and 4a), viral DNA synthesis (Figs 3b and 4b), progeny production (Figs 3c and 4c and Supplementary Figure 1) or extracellular particle EBV DNA synthesis (Fig. 3d), at least in our HEK293 system. Knockdown of BRRF1 also did not affect viral protein expression, viral DNA synthesis and progeny production (Supplementary Figure 2). A previous study of EBV BRRF1 used a BRLF1 and BRRF1 double-knockout mutant virus, whereby approximately 1,400 nts of the BRLF1 gene were replaced with a tetracycline resistance cassette^13^. The results showed that BRRF1 enhanced BRLF1-induced reactivation in HEK293 cells and gastric carcinoma cells, but not in lymphoblastoid or Burkitt cell lines. However, we did not observe any significant difference between wild-type and mutant viruses in HEK293 cells. Because the previous study used a cassette insertion mutant virus for phenotype analysis, expression of neighbouring genes, such as BRRF2, may have been affected, as we experienced (Fig. 3a). Importantly, exogenous complement of BRLF1 and BRRF2, along with BZLF1, could rescue the reduced expression of gB and lytic LMP1, and EBV DNA replication in our insertion mutant (dBRRF1ins) (Supplementary Figure 3). Therefore, the results from the previous study may have been due to defects in neighbouring genes. In another study from the same group, knockdown of endogenous BRRF1 expression inhibited chemically induced lytic reactivation in nasopharyngeal carcinoma cell lines^12^. The discrepancy between our results and that study may be due to off-target effects or differences in cell line used.

The BRRF1 gene is a positional homolog of ORF49 from Kaposi’s sarcoma-associated herpesvirus (KSHV), murine gammaherpesvirus 68 (MHV-68) and herpesvirus saimiri, even though amino acid sequence similarity between these proteins is not very high. In contrast to our results, ORF49 proteins of KSHV and MHV-68 were reported to functionally cooperate with RTA, a homologue of the EBV BRLF1 gene^16, 17^. Differences in gene functions between EBV and KSHV or MHV-68 are not rare, because EBV is a gamma1 herpesvirus, while KSHV and MHV-68 are both gamma2 herpesviruses.

Since a study on MHV-68 ORF49 suggested that the virion-associated ORF49 protein might promote viral replication by providing an optimal environment during the early phase of viral infection^18^, we evaluated pre-latent abortive lytic gene expression using Akata(-) cells and PBMCs. However, the transcript levels of IE, E, L and latent genes in the dBRRF1 virus were comparable to those in the wild-type and dBRRF1/R viruses (Fig. 5a,b).

The transforming efficacy of the BRRF1-deficient virus was comparable to those of the wild-type and revertant viruses (Fig. 5c). The growth of LCLs induced by the BRRF1-deficient virus was also comparable to that of the wild-type and revertant viruses (Fig. 5d). Taken together, these results indicate that BRRF1 is dispensable for viral replication in HEK293 cells and B cell transformation at cell culture levels.

A previous study utilizing a CAT assay in HeLa cells reported that BRRF1 activated the BZLF1 promoter through a CRE motif by enhancing the transcriptional activity of c-Jun, which forms AP-1 in combination with c-Fos^13^. Here, we also observed in HeLa cells that BRRF1 had a positive effect on BZLF1 promoter activity (Fig. 6a), but the effect was not very strong, and BRRF1 did not increase BZLF1 promoter activity in AGS cells (Fig. 6d). This finding suggests that BRRF1 might act in a cell-type-dependent manner on the BZLF1 promoter. The CRE-, AP-1- and NFκB-dependent promoters were activated to some extent by BRRF1 in AGS and HeLa cells in our luciferase assays (Fig. 6b,e). The p53-mediated transcription was also activated by BRRF1 (Fig. 7), although a previous report showed no activation of p53 response elements by BRRF1 in reporter assays^12^.

Since it was reported that BRRF1 and p53 induced lytic protein expression synergistically in nasopharyngeal cells harboring EBV^12^, we performed forced expression analysis using p53, BRRF1, BZLF1 and BRLF1 expression vectors in HEK293 cells harboring wild-type EBV BAC DNA (Fig. 8). Although BRRF1 marginally induced expression of a subset of viral genes, it did not further enhance the lytic protein expression induced by either BZLF1 or BRLF1, in contrast to a previous report^12^ (Fig. 8). EBV DNA replication was not induced by BRRF1 with or without BZLF1 or BRLF1 (Fig. 8). Again, this result indicates that BRRF1 does not notably affect the viral lytic replication cycle in HEK293 cells.

Recently, the KSHV ORF49 protein, a BRRF1 homologue, was suggested to interact and bind to DNA^19^. Furthermore, BRRF1 was reported to influence EBI2 expression in B lymphocytes directly^20^. In light of these studies, our results indicate that BRRF1 may affect host transcription, but its effect on EBV lytic replication may not be very strong.

We report for the first time that the BRRF1 gene is dispensable for EBV lytic replication, at least in HEK293 cells, and B cell transformation at the cell culture level. Further studies are required to clarify the role of the BRRF1 gene in EBV lytic replication. For example, the development of gene-editing methods using the CRISPR/Cas9 system for viral mutagenesis may facilitate functional analysis of the gene product in the future. *In vivo* studies, such as a humanized mouse model, may also provide significant data on the phenotype induced by the BRRF1 gene.

## Methods

### Cell culture and reagents

HEK293, HEK293T and HeLa cells were maintained in Dulbecco’s Modified Eagle’s Medium (Sigma) containing 10% foetal bovine serum (FBS). B95-8, Akata(-), AGS-Akata and AGS(-) cells and LCLs were cultured in RPMI1640 medium containing 10% FBS. PBMCs were cultured in RPMI1640 medium containing 10% FBS, ciclosporin A (1 μg/ml) and 1% non-essential amino acid solution. TPA, A23187, PAA and non-essential amino acid solution were purchased from Sigma. Sodium butyrate and ciclosporin A were purchased from Wako chemicals. The antibody against BRRF1 was prepared by immunizing a rabbit with a fusion protein of GST and full-length BRRF1. Antibodies against BRRF2, BZLF1, BMRF1, BALF2, gB, EBNA1 and LMP1 were used as described previously^14, 21–23^. Antibodies against tubulin, BRLF1 and p53 were purchased from Cell Signaling, Argene Biosoft and Merk, respectively. Horseradish peroxidase-linked goat antibodies to mouse or rabbit IgG were purchased from Amersham Biosciences. Hygromycin B was purchased from Clontech.

PBMCs were obtained from a healthy adult donor according to protocols approved by the Institutional Review Board of Nagoya University Hospital. Informed consent was obtained from the donor, and all methods were performed considering minimal invasiveness, anonymization, strict control of specimens and information, according to the Declaration of Helsinki.

### Plasmid construction

The expression vector pCMVp53 was obtained from T. Takahashi^24^. The expression vector pcDNABZLF1^22^ and pcDNABRRF2^23^ were constructed as described previously. The BRRF1 PCR product was cloned into a pcDNA3 vector to generate the BRRF1 expression plasmid. The primer sequences used for BRRF1 amplification were 5′-ATCT**GAATTC**ATGGCTAGTAGTAACAGAGG-3′ (forward) and 5′-TA**GCGGCCGC**TTATTTGTATTGCATGGCAG-3′ (reverse), where bold sequences represent EcoRI and NotI recognition motifs, respectively. To generate the BRLF1 expression plasmid, the coding sequences of the B95-8 BRLF1 gene were amplified by PCR, and the PCR product was cloned into a pcDNA3.1(-) vector after digestion with HindIII and EcoRI.

### EBV-BAC DNA construction

EBV-BAC DNA was obtained from W. Hammerschmidt^25^. Homologous recombination was performed in *Escherichia coli* as described previously^14, 22^. A marker cassette (Neo/St cassette), which contains neomycin resistance and streptomycin sensitivity genes, in rpsL-neo (Gene Bridges) was used to manipulate recombinant viruses. PCR fragments used for the manipulation were prepared using a template, the rpsL-neo vector and the following primers: 5′-ATGGCTAGTAGTAACAGAGGAAATGCCCGACCATTAAAATCTTTCCTCCATGAGCTTTACggcctggtgatgatggcgggatc-3′ (forward) and 5′-TGGCTAGGTGGGAGGTCGCAGTCGACCCCGATGGTGTTCAGT AGATGCACCACATCCCCCtcagaagaactcgtcaagaagg-3′ (reverse), where capital letters represent EBV BRRF1 gene sequences. Recombinant dBRRF1 viruses were constructed using PCR fragments amplified by the following primers: 5′-CTAAGTGGGGGATGTGGTGC-3′ (forward) and 5′-GGTTAGTGTTTCAGGTAAAGCTC-3′ (reverse). Recombinant dBRRF1/R and dBRRF1ins/R viruses were generated using PCR fragments amplified by the following primers: 5′-ATGGCTAGTAGTAACAGAGG-3′ (forward) and 5′-TGCTGTACAGCCTGCAAGAC-3′ (reverse). Before and after introduction of the recombinants into HEK cells, PCR was performed to confirm the sequences (approximately 200 nts around the mutated nts) of the recombinants using the following primers: 5′-TAGCCTCAGAAAGTCTTCCA-3′ (forward) and 5′-TGCTGTACAGCCTGCAAGAC-3′ (reverse). Using these primers, the sequences of approximately 200 nts around the stop codon introduced into the dBRRF1 strain were confirmed. Electroporation was performed using the Gene Pulser III (Bio-Rad), and EBV-BAC DNA was purified using NucleoBond Bac100 (Macherey-Nagel).

### Transfection of EBV-BAC DNA and cell cloning

Recombinant EBV-BAC DNA was transfected into HEK293 cells by lipofection using Lipofectamine 2000 (Invitrogen). Transfected HEK293 cells were cultured with 150 μg/ml hygromycin B, and colonies with high GFP expression were cloned as described previously^22^. Clones expressing fewer lytic proteins in the latent state and more lytic proteins after induction by BZLF1 were selected. The purity of each EBV-BAC cell line was confirmed by sequencing.

### Immunoblotting

HEK293 cells harboring each recombinant EBV were transfected with 1 μg pcDNABZLF1, pcDNABRLF1, pcDNABRRF1 or pCMVp53 using the Neon Transfection System (Invitrogen) to induce lytic replication. Two days after BZLF1, BRRF1 or p53 transfection and 3 days after BRLF1 transfection, cells were collected for protein analysis after washing with PBS. Immunoblotting was performed using anti-BZLF1, -BRLF1, -BALF2, -BMRF1, -BRRF1, -BRRF2, -gB, -EBNA1, -LMP1, -p53 and -tubulin antibodies as described previously^22^.

### Quantification of EBV DNA synthesis

HEK293 cells infected with latent EBV were harvested at the indicated points, and viral DNA levels were analysed by real-time PCR using the Mx3000 P qPCR system (Agilent Technologies) as described previously^26^. The intensity of the Rox dye, FastStart Universal Probe Master (Rox) (Roche Applied Science), was used to compensate for volume differences among the tubes. Eukaryotic 18 S rRNA (Applied Biosystems) was used as an endogenous control. For EBV genome detection, primers and a probe targeting the BALF2-coding region were used as described previously^23^.

### Determination of progeny virus titres

After a freeze-thaw process and centrifugation, each virus stock solution was co-cultured with Akata(-) cells. After 2 days, cells were fixed with 1% formaldehyde and suspended in PBS. GFP-positive cells, representing the progeny virus titres, were counted using the FACS Calibur G5 system (Becton-Deckinson).

### RNA interference

Control siRNA and siRNAs against BRRF1 were purchased from Gene Design, Inc. The sequences of siRNAs were as follows: for siCtrl, 5′-GCAGAGCUGGUUUAGUGAATT-3′ (sense) and 5′-UUCACUAAACCAGCUCUGCTT-3′ (antisense), for siBRRF1-1, 5′-GCUUUACCUGAAACACUAUTT-3′ (sense) and 5′-AUAGUGUUUCAGGUAAAGCTT-3′ (antisense), for siBRRF1-2, 5′-GAUGCAUUUACUAUAACUATT-3′ (sense) and 5′-UAGUUAUAGUAAAUGCAUCTT-3′ (antisense), for siBRRF1-3, 5′-CUACAUUCAGGGAUCUAUATT-3′ (sense) and 5′-UAUAGAUCCCUGAAUGUAGTT-3′ (antisense). Quantification of the immunoblot bands was performed using Image J.

### Quantification of extracellular virion EBV DNA

Two hundred μl of each supernatant obtained from HEK293 cells after BZLF1 expression was treated with 2 μl of Turbo DNaseI (Ambion), and then inactivated by heat shock at 75 °C and EDTA, as described previously^27^. After DNA purification using the DNeasy kit (QIAGEN), EBV and the host genome DNA levels were quantified by qPCR as described above.

### Viral mRNA analysis during lytic replication

B95-8 cells were lytically induced by 200 ng/ml TPA, 0.5 μM A23187 and 5 mM sodium butyrate with or without 400 μg/ml PAA for 48 hours^23^. AGS-Akata cells were lytically induced by 10 ng/ml TPA and 2 mM sodium butyrate with or without 200 μg/ml PAA for 48 hours. After treatment, cells were washed with PBS and collected. Total RNA was purified using TriPure Isolation Reagent (Roche), and the levels of viral mRNA were determined by qRT-PCR using the One Step SYBR PrimeScript RT-PCR Kit II (TaKaRa) and the Mx3000 P qPCR system (Agilent Technologies), as described previously^14^. The primer sequences used for qRT-PCR were as follows: for BALF2, 5′-GGGCTGTGGCGAGTACCAC-3′ and 5′-CGCTGGTCCTGTGTGTCTTG-3′, for BMRF1, 5′-TTAGAAACCTTGCCTACGGG-3′ and 5′-AAAATTGCAGGGAAGCCTGC-3′, for BRRF1, 5′-AAGAGTCTTGCAGGCTGTAC-3′ and 5′-GAGTAGTAGCTTAGCAGCTC-3′, for MCP, 5′-AGGTTGGGAGGAAAACGTAG-3′ and 5′-TTAACGGAGACCACGACCAC-3′, for gp350, 5′-CCCTCACTACTGCCGTTATA-3′ and 5′-GCCTGGAATCTGTAGATGTC-3′, for GAPDH, 5′-TGCACCACCAACTGCTAGC-3′ and 5′-GGCATGGACTGTGGTCATGAG-3′.

### Viral mRNA analysis during the pre-latent abortive lytic cycle

The supernatants of HEK293 cells after induction by BZLF1, which were normalized to the equal infectious titre, were co-cultured with Akata(-) cells or PBMCs. After 48 hours, cells were washed with PBS and collected. Total RNA was purified, and viral mRNA levels were determined by qRT-PCR, as described above. The primers used for detection of BZLF1 and BRLF1 were the same as described previously^28^. The primers for BRRF1, BALF2 and BMRF1 were the same as described above. The remaining primers were as follows: for BHRF1, 5′-TGGCCTATTCAACAAGGGAG-3′ and 5′-TTTCTCTTGCTGCTAGCTCC-3′, for gB, 5′-TCACCTGCTCTTCGATGCAC-3′ and 5′-GCGCAAACGGACTCAACGTG-3′, for LMP1, 5′-CTATTCCTTTGCTCTCATGC-3′ and 5′-TGAGCAGGAGGGTGATCATC-3′, for LMP2, 5′-AGATCCTTCTGGCACGACTG-3′ and 5′-CATGCAGAACAAATTGGGTATA-3′, for EBNA1, 5′-AGGTACAGGACCTGGAAATG-3′ and 5′-CCTCGTCCATGGTTATCACC-3′, for EBNA2, 5′-TTAGAGAGTGGCTGCTACGCATT-3′ and 5′-TCACAAATCACCTGGCTAAG-3′, for EBNA3c, 5′-CAAGAGATCAGCAACCTTGG-3′ and 5′-GGTTTGATAGCGCTTGCTTG-3′.

### Growth transformation assay

Serial 10-fold dilutions of each recombinant virus, which were normalized to the equal infectious titre, were co-cultured with 1 × 10^5^ PBMCs in 96-well plates. Every 5 days, half of the medium was replaced with fresh medium. The number of wells with LCLs was counted at 25 days after infection, and the 50% transforming doses were calculated using the Spearman-Karber method^29^. After expansion, LCLs were seeded at 1 × 10^5^/ml and counted after 2, 4, 5 and 7 days.

### Luciferase assay

Plasmid DNA was transfected into HeLa, AGS(-) or HEK293T cells using Lipofectamine 2000 (Invitrogen). The total amount of plasmid DNA for each sample was normalized by adding pcDNA3 empty vector. After 24 hours, cells were treated with passive lysis buffer (Promega), and luciferase activity was analysed. Fold activation of firefly luciferase was calculated after normalization to those of renilla luciferase. The protein samples were analysed by immunoblotting. The pBZLF1-FL and pBALF2-FL reporter plasmids have been described previously^30, 31^. The pCRE-FL, pNF-κB-FL, pAP1-FL, pCMV-RL and null-RL (pGL4.70) reporter plasmids were purchased from Promega. The PG13-Luc and MG15-Luc plasmids were donated by Dr. B. Vogelstein^32^.

## Electronic supplementary material

Supplementary Information

## References

[1] Murata T, Tsurumi T (2014). Switching of EBV cycles between latent and lytic states. Rev Med Virol..

[2] Young LS, Rickinson AB (2004). Epstein-Barr virus: 40 years on. Nat Rev Cancer..

[3] Murata T, Sato Y, Kimura H (2014). Modes of infection and oncogenesis by the Epstein-Barr virus. Rev Med Virol..

[4] Murata T (2014). Regulation of Epstein-Barr virus reactivation from latency. Microbiol Immunol..

[5] Murata T, Tsurumi T (2013). Epigenetic modification of the Epstein-Barr virus BZLF1 promoter regulates viral reactivation from latency. Front Genet..

[6] Westphal EM, Blackstock W, Feng W, Israel B, Kenney SC (2000). Activation of lytic Epstein-Barr virus (EBV) infection by radiation and sodium butyrate *in vitro* and *in vivo*: a potential method for treating EBV-positive malignancies. Cancer Res..

[7] Sinclair AJ (2003). bZIP proteins of human gammaherpesviruses. J Gen Virol..

[8] Zalani S, Holley-Guthrie E, Kenney S (1996). Epstein-Barr viral latency is disrupted by the immediate-early BRLF1 protein through a cell-specific mechanism. Proc Natl Acad Sci USA.

[9] Sugimoto A (2013). Different distributions of Epstein-Barr virus early and late gene transcripts within viral replication compartments. J Virol..

[10] Kalla M, Hammerschmidt W (2012). Human B cells on their route to latent infection–early but transient expression of lytic genes of Epstein-Barr virus. Eur J Cell Biol..

[11] Segouffin-Cariou C, Farjot G, Sergeant A, Gruffat H (2000). Characterization of the epstein-barr virus BRRF1 gene, located between early genes BZLF1 and BRLF1. J Gen Virol..

[12] Hagemeier SR, Barlow EA, Kleman AA, Kenney SC (2011). The Epstein-Barr virus BRRF1 protein, Na, induces lytic infection in a TRAF2- and p53-dependent manner. J Virol..

[13] Hong GK (2004). The BRRF1 early gene of Epstein-Barr virus encodes a transcription factor that enhances induction of lytic infection by BRLF1. J Virol..

[14] Watanabe T (2015). The Epstein-Barr Virus BDLF4 Gene Is Required for Efficient Expression of Viral Late Lytic Genes. J Virol..

[15] Yuan J, Cahir-McFarland E, Zhao B, Kieff E (2006). Virus and cell RNAs expressed during Epstein-Barr virus replication. J Virol..

[16] Gonzalez CM (2006). Identification and characterization of the Orf49 protein of Kaposi’s sarcoma-associated herpesvirus. J Virol..

[17] Lee S (2007). The ORF49 protein of murine gammaherpesvirus 68 cooperates with RTA in regulating virus replication. J Virol..

[18] Noh CW (2012). The virion-associated open reading frame 49 of murine gammaherpesvirus 68 promotes viral replication both *in vitro* and *in vivo* as a derepressor of RTA. J Virol..

[19] Hew, K. *et al*. Structure of the Open Reading Frame 49 Protein Encoded by Kaposi’s Sarcoma-Associated Herpesvirus. *J Virol*. **91** (2017). 10.1128/JVI.01947-16 PMC5215346 27807232

[20] Cornaby C (2017). EBI2 expression in B lymphocytes is controlled by the Epstein-Barr virus transcription factor, BRRF1 (Na), during viral infection. J Gen Virol..

[21] Daikoku T (2005). Architecture of replication compartments formed during Epstein-Barr virus lytic replication. J Virol..

[22] Murata T (2009). Efficient production of infectious viruses requires enzymatic activity of Epstein-Barr virus protein kinase. Virology..

[23] Watanabe T (2015). The Epstein-Barr virus BRRF2 gene product is involved in viral progeny production. Virology..

[24] Takahashi T (1992). Wild-type but not mutant p53 suppresses the growth of human lung cancer cells bearing multiple genetic lesions. Cancer Res..

[25] Delecluse HJ, Hilsendegen T, Pich D, Zeidler R, Hammerschmidt W (1998). Propagation and recovery of intact, infectious Epstein-Barr virus from prokaryotic to human cells. Proc Natl Acad Sci USA.

[26] Narita Y (2013). Pin1 interacts with the Epstein-Barr virus DNA polymerase catalytic subunit and regulates viral DNA replication. J Virol..

[27] Wang X (2015). Mono-ubiquitylated ORF45 Mediates Association of KSHV Particles with Internal Lipid Rafts for Viral Assembly and Egress. PLoS Pathog..

[28] Murata T (2013). Contribution of myocyte enhancer factor 2 family transcription factors to BZLF1 expression in Epstein-Barr virus reactivation from latency. J Virol..

[29] Finney, D. J. *Stastistical Methods in Biological Assay*. 2nd edn, 524–533 (Charles Griffin & Company Limited, 1952).

[30] Murata T (2009). TORC2, a coactivator of cAMP-response element-binding protein, promotes Epstein-Barr virus reactivation from latency through interaction with viral BZLF1 protein. J Biol Chem..

[31] Nakayama S (2009). Epstein-Barr virus polymerase processivity factor enhances BALF2 promoter transcription as a coactivator for the BZLF1 immediate-early protein. J Biol Chem..

[32] el-Deiry WS (1993). WAF1, a potential mediator of p53 tumor suppression. Cell..

