# Supplementary material for: The Epstein-Barr Virus BRRF1 Gene Is Dispensable for Viral Replication in HEK293 cells and Transformation
Source: Sci Rep. 2017 Jul 20;7:6044. doi: 10.1038/s41598-017-06413-7 (PMC5519699; doi:10.1038/s41598-017-06413-7)
Supplement: Supplementary file 1 — Supplementary Information [file 41598_2017_6413_MOESM1_ESM.pdf]

Supplementary Information

The Epstein-Barr Virus BRRF1 Gene Is Dispensable for Viral Replication in HEK293 cells and Transformation

Masahiro Yoshida, Takahiro Watanabe, Yohei Narita, Yoshitaka Sato, Fumi Goshima, Hiroshi Kimura, and Takayuki Murata*

Department of Virology, Nagoya University Graduate School of Medicine, 65 Tsurumai-cho, Showa-ku, Nagoya 466-8550, Japan

Supplementary Figure 1

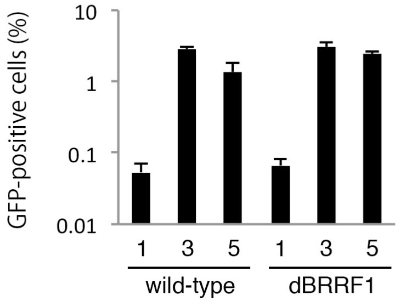

Time course of progeny production in wild-type and dBRRF1 viruses after forced expression of BZLF1. HEK293 cells harboring the EBV BAC were transfected with the BZLF1 expression vector. Viruses were collected from the media and cells on days 1, 3 and 5. After a freeze-thaw process and centrifugation, supernatants were cultured with Akata(-) cells. GFP-positive cells, representing the progeny virus titres, were counted using FACS. Each bar represents the mean and standard deviation of three independent experiments.

Supplementary Figure 2

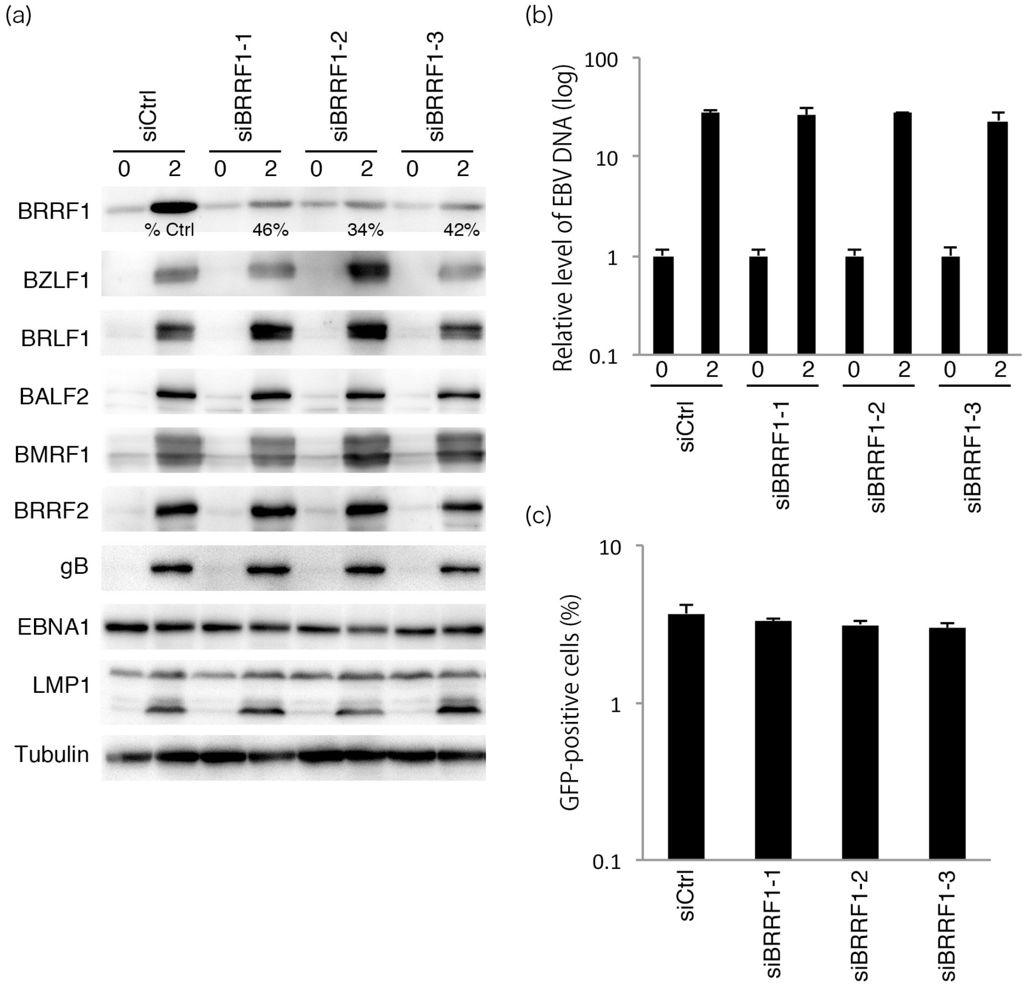

Lytic protein expression, EBV DNA synthesis and progeny production in wild-type viruses after transfection of BZLF1 expression vector and siRNAs. (a) HEK293 cells harboring the wild-type EBV BAC were transfected with the BZLF1 expression vector and control siRNA or siRNAs against BRRF1. Cells were collected on days 0 and 2 and subjected to immunoblotting. Numbers below the BRRF1 bands indicate the levels of BRRF1 expression normalized to those of tubulin. The original blots are presented in Supplementary Figure 4 and 5. (b) Cells were collected on days 0 and 2 and subjected to qPCR. Each bar represents the mean and standard deviation of three independent experiments. (c) Viruses were collected from the media and cells on day 3. After a freeze-thaw process and centrifugation, supernatants were cultured with Akata(-) cells. GFP-positive cells, representing the progeny virus titres, were counted using FACS. Each bar represents the mean and standard deviation of three independent experiments.

Supplementary Figure 3

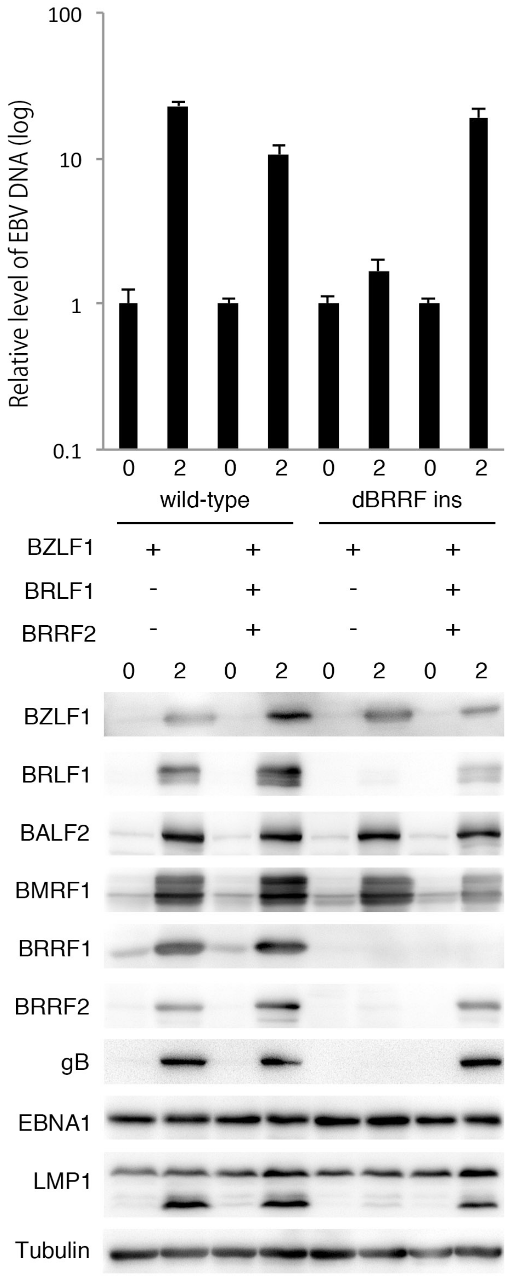

Lytic protein expression and EBV DNA synthesis in wild-type and dBRRF1ins viruses after transfection of BZLF1 expression vector with or without BRLF1 and BRRF2 expression vectors. HEK293 cells harboring the EBV BAC were transfected with the BZLF1 expression vector with or without BRLF1 and BRRF2 expression vectors. Cells were collected on days 0 and 2 and subjected to immunoblotting and qPCR. The original blots are presented in Supplementary Figure 6 and 7. EBV DNA levels were analysed by qPCR. Each bar represents the mean and standard deviation of three independent experiments.

Supplementary Figure 4

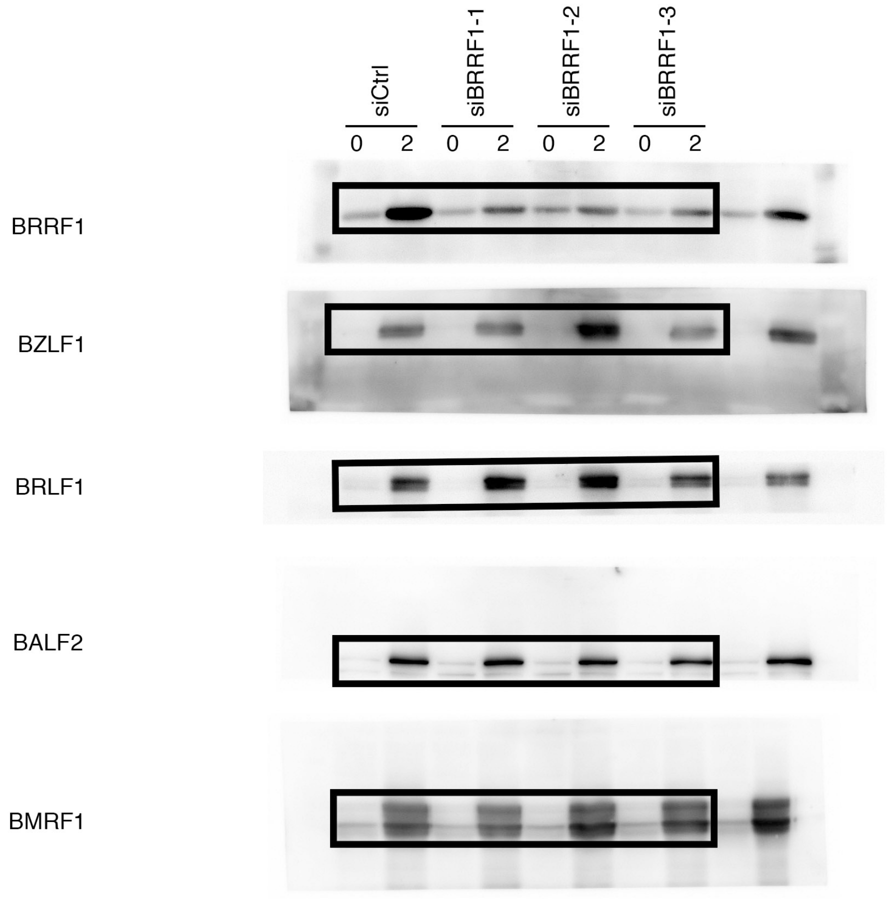

Images surrounded by ☐ were used for Supplementary Fig. 2

Supplementary Figure 5

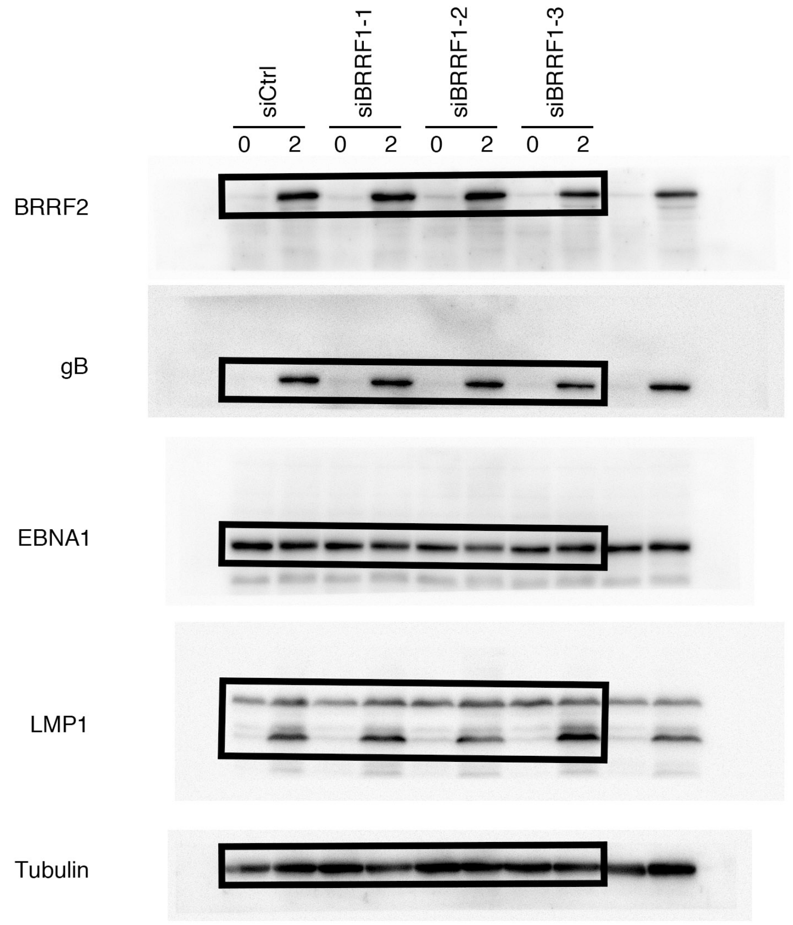

Images surrounded by ☐ were used for Supplementary Fig. 2

Supplementary Figure 6

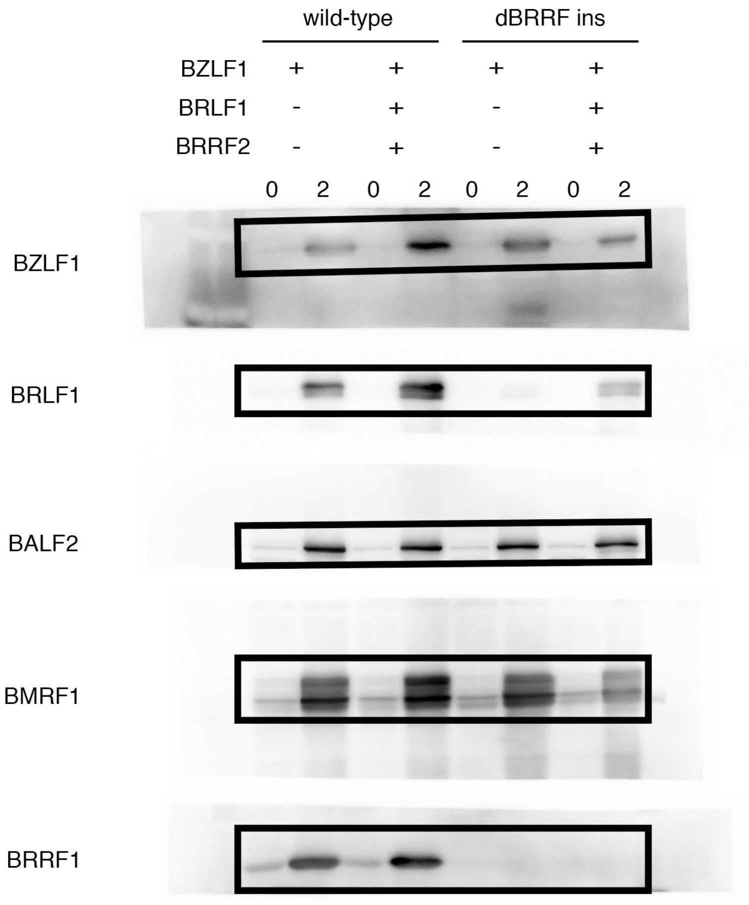

Images surrounded by ☐ were used for Supplementary Fig. 3

Supplementary Figure 7

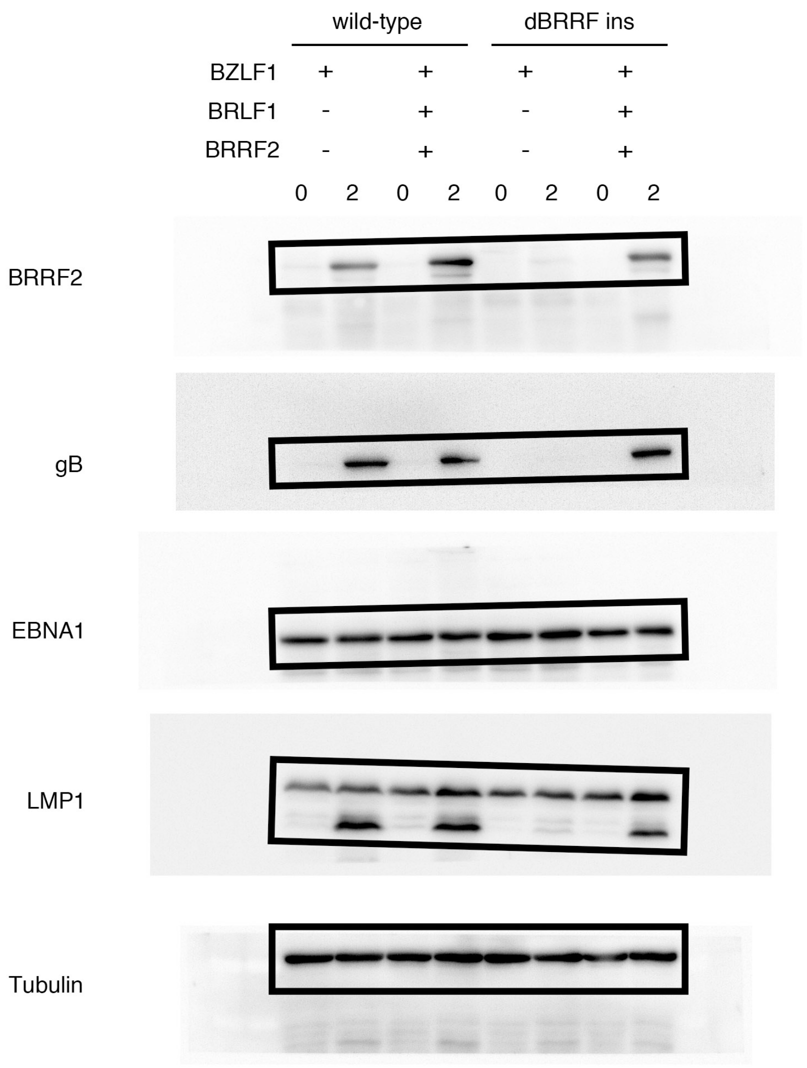

Images surrounded by ☐ were used for Supplementary Fig. 3

Supplementary Figure 8

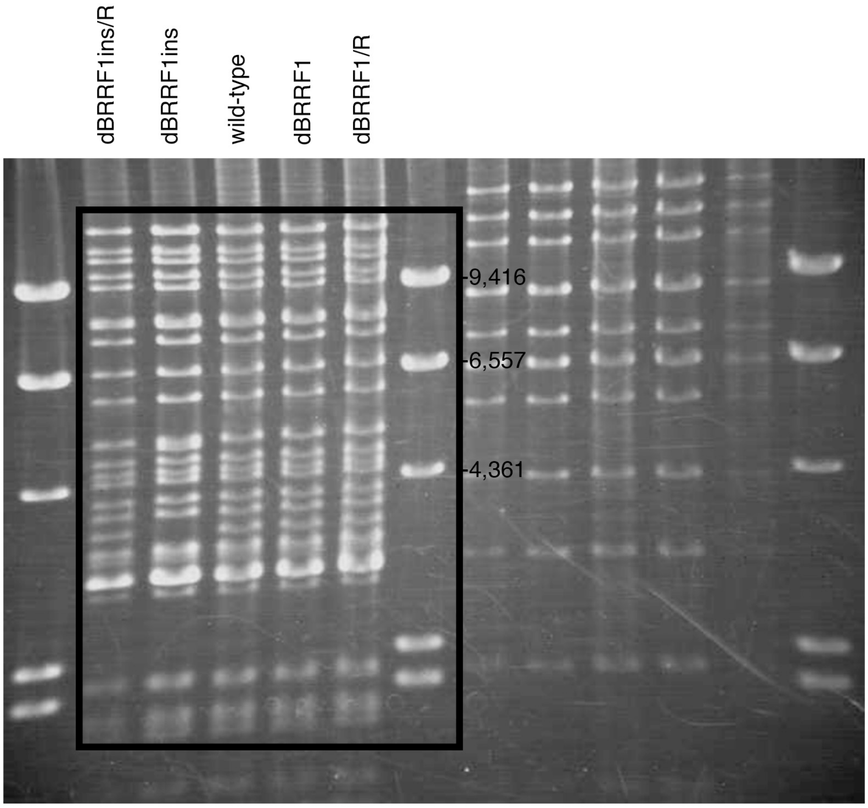

BamHI

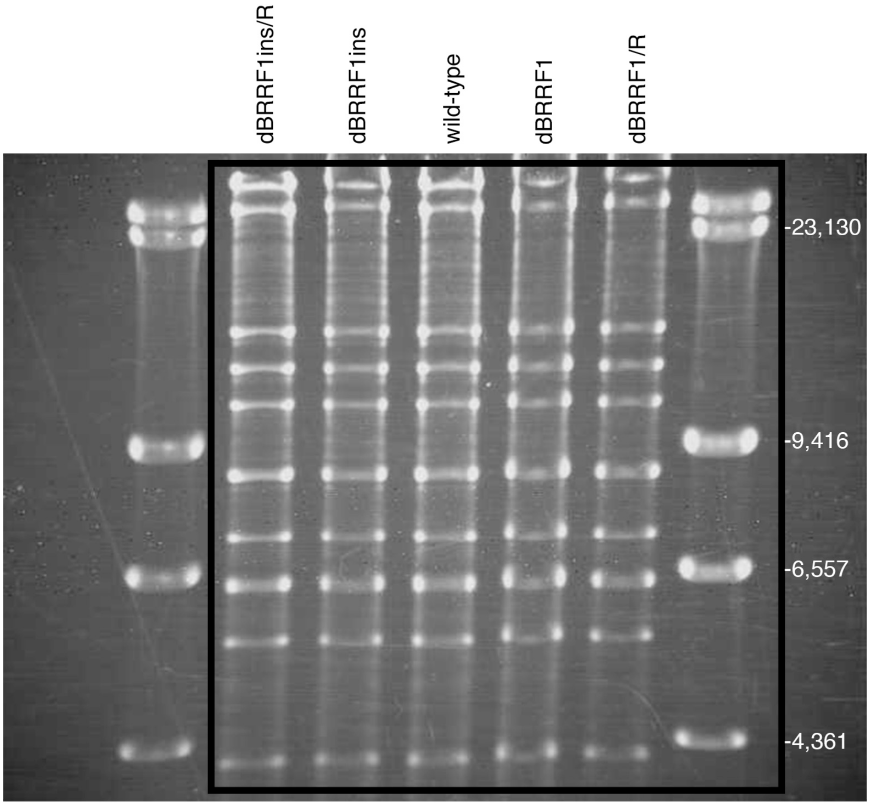

EcoRI

Images surrounded by □ were used for Fig. 2b

Supplementary Figure 9

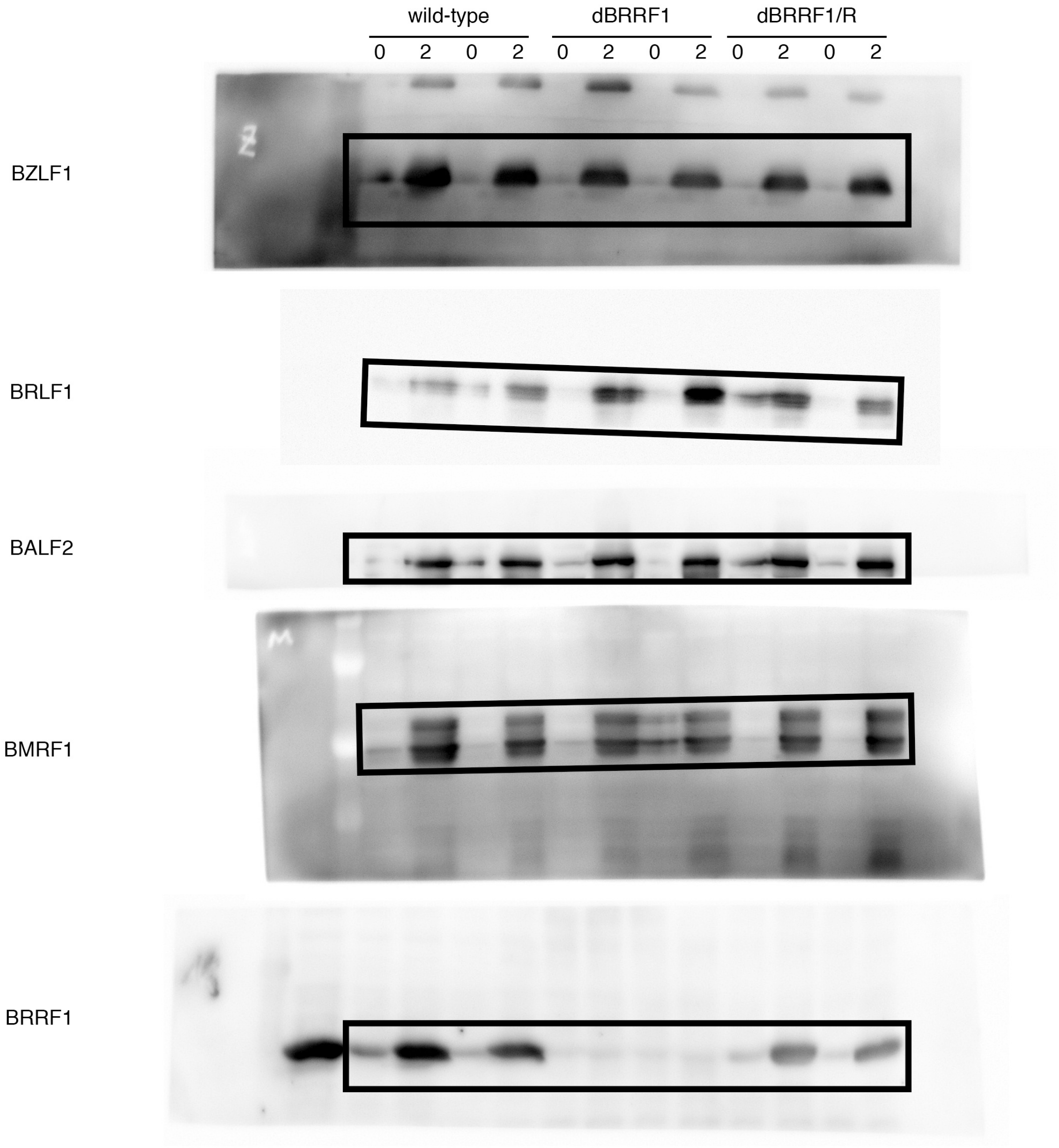

Images surrounded by ☐ were used for Fig. 3a

Supplementary Figure 10

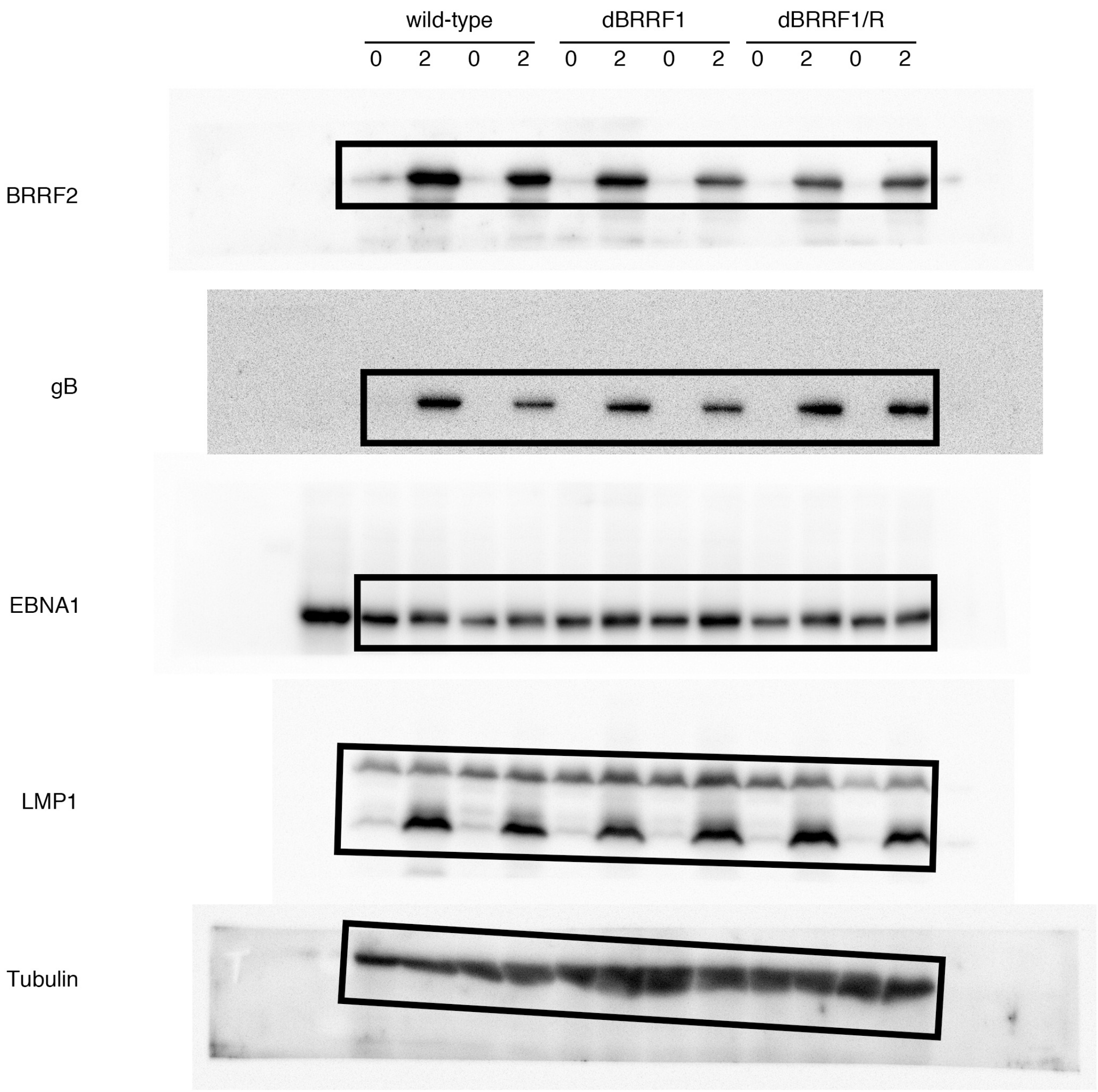

Images surrounded by □ were used for Fig. 3a

Supplementary Figure 11

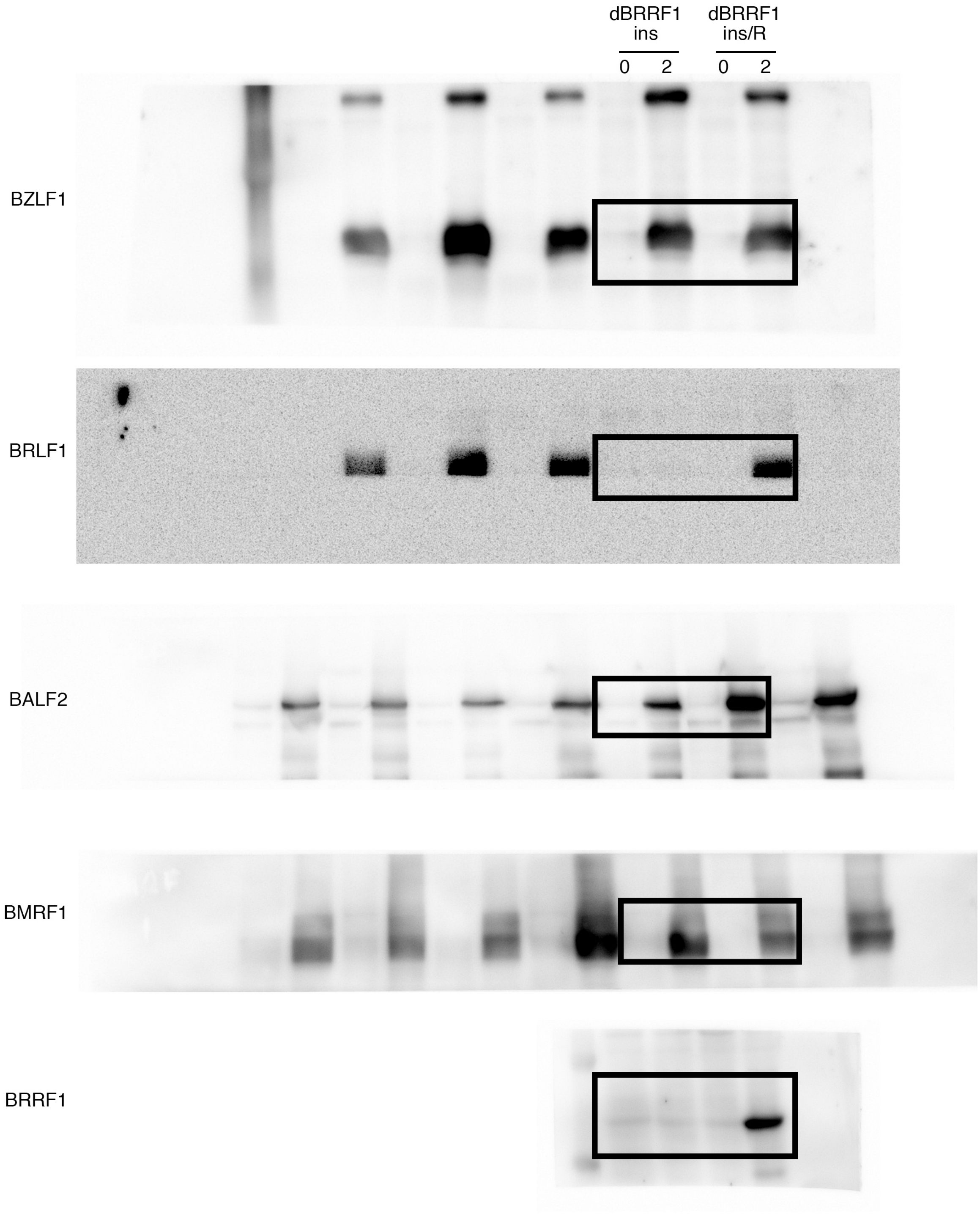

Images surrounded by □ were used for Fig. 3a

Supplementary Figure 12

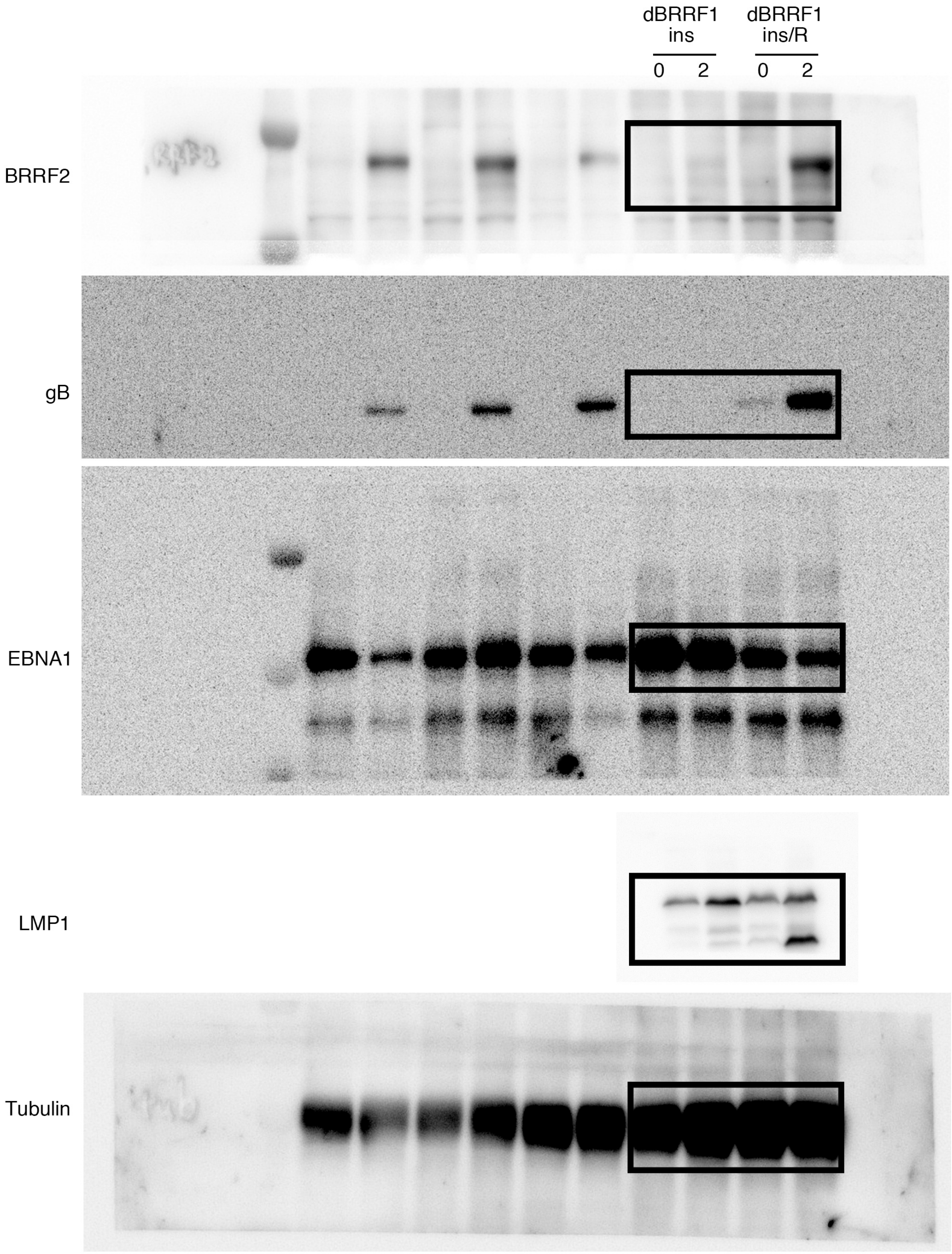

Images surrounded by ☐ were used for Fig. 3a

Supplementary Figure 13

Representative FACS results of Figure 3c.

R2 gated region indicates GFP-positive cells.

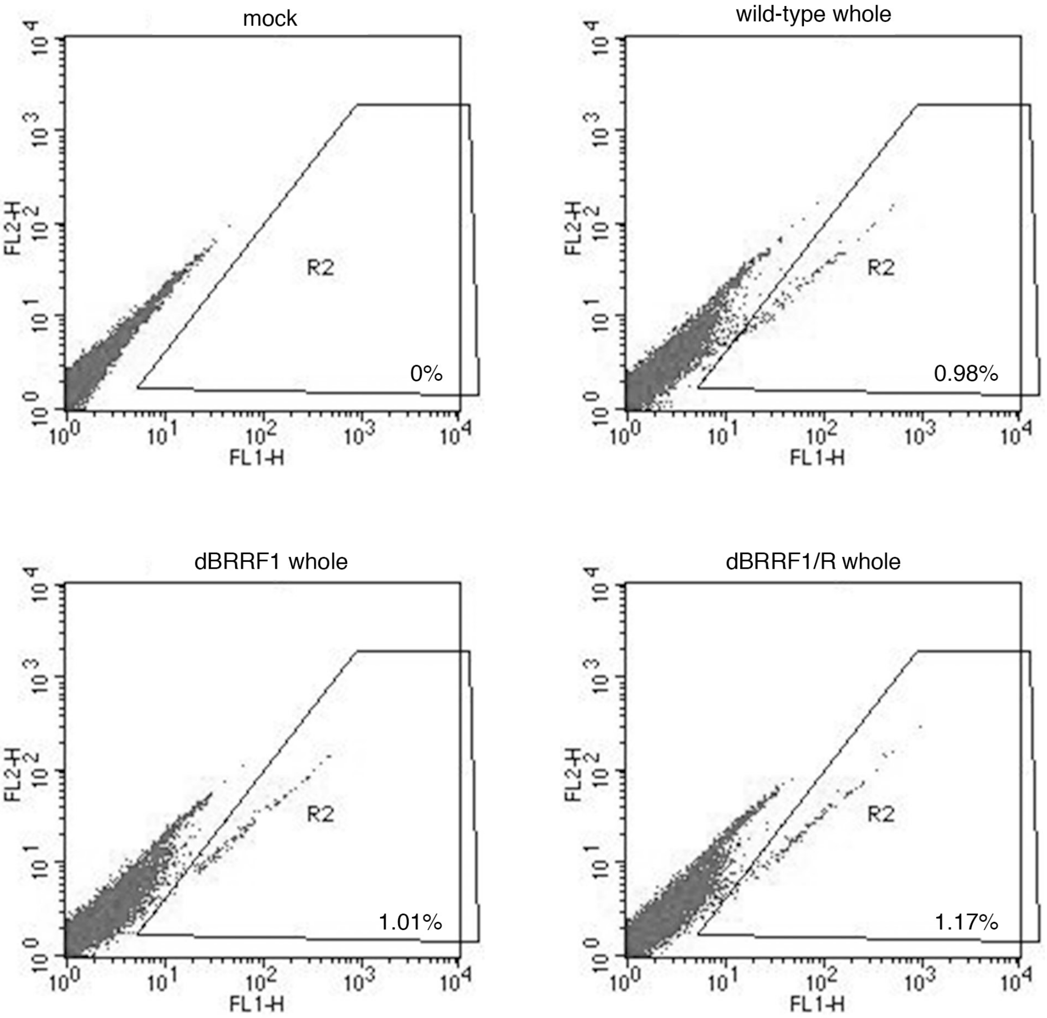

Supplementary Figure 14

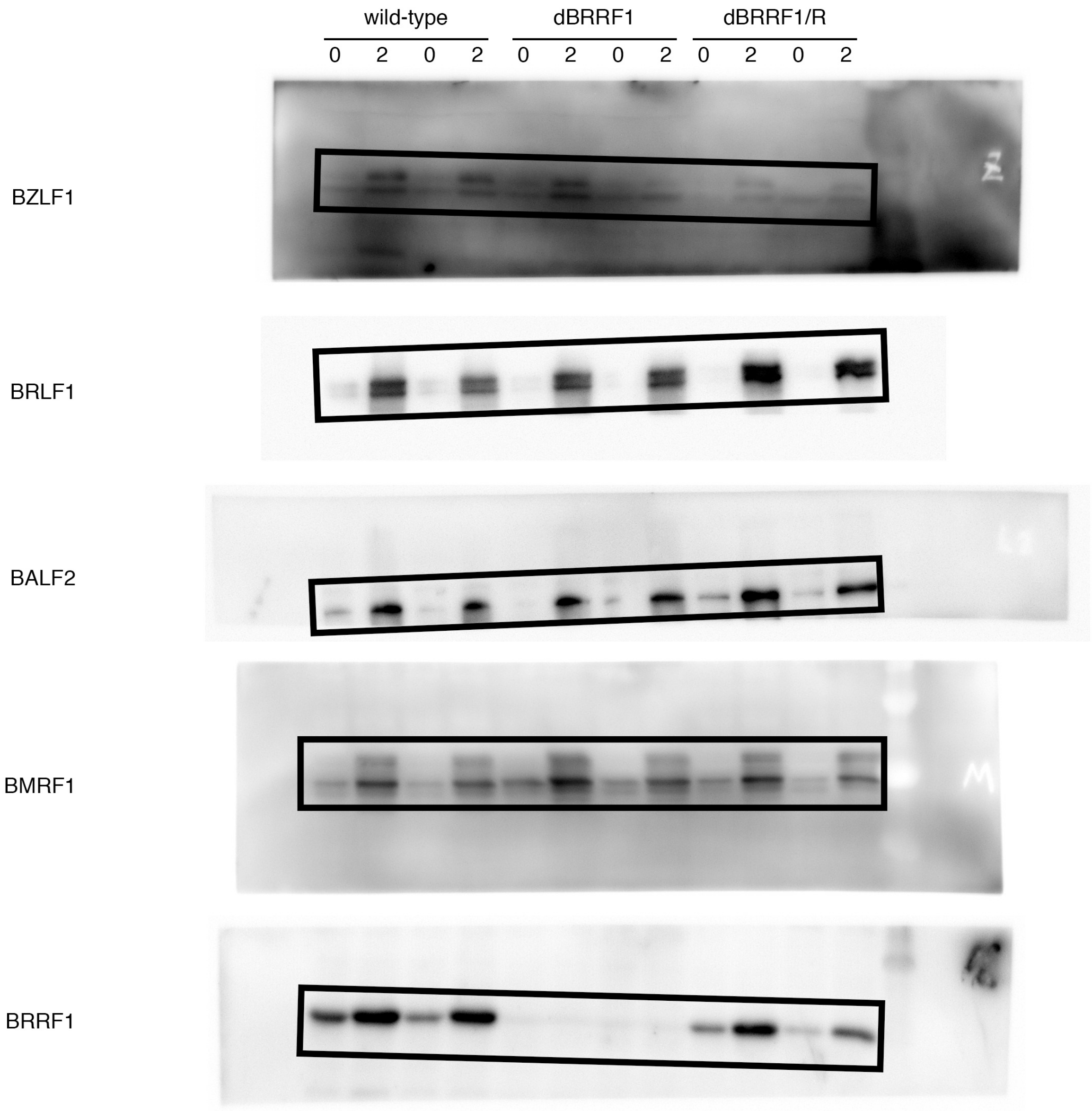

Images surrounded by □ were used for Fig. 4a

Supplementary Figure 15

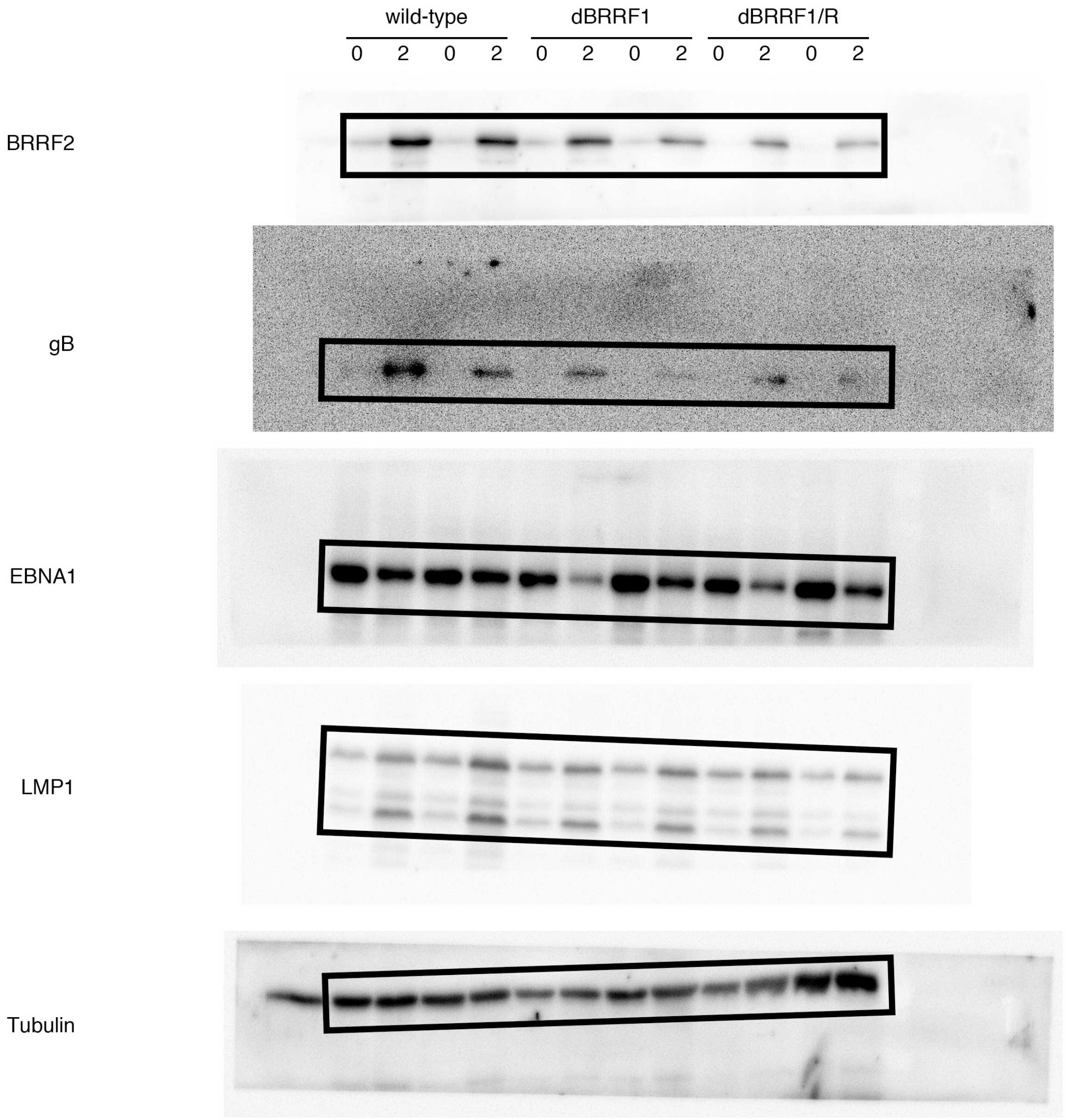

Images surrounded by □ were used for Fig. 4a

Supplementary Figure 16

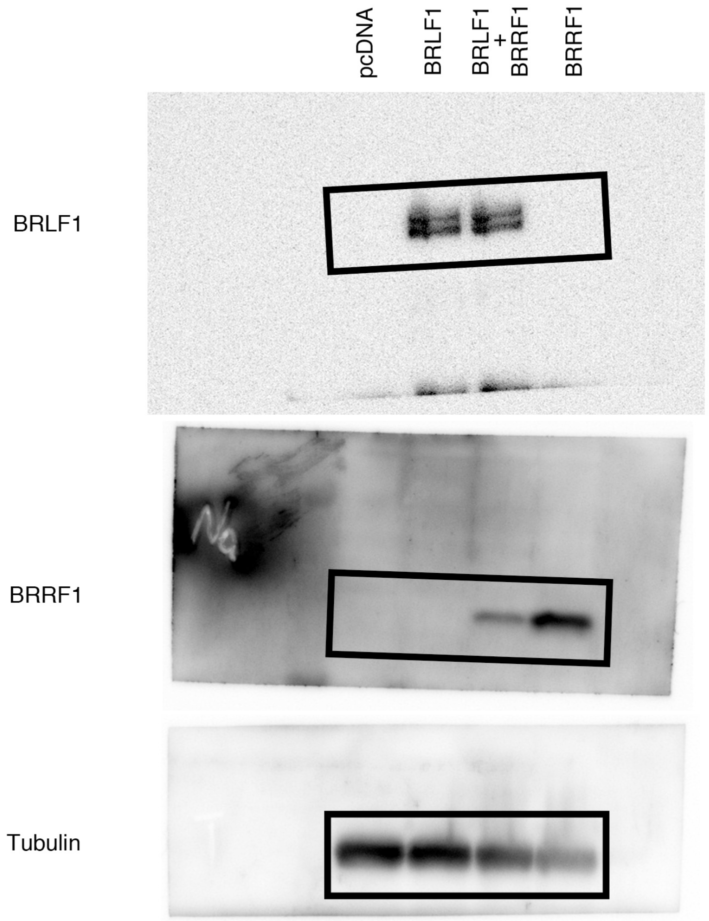

Images surrounded by □ were used for Fig. 6a

Supplementary Figure 17

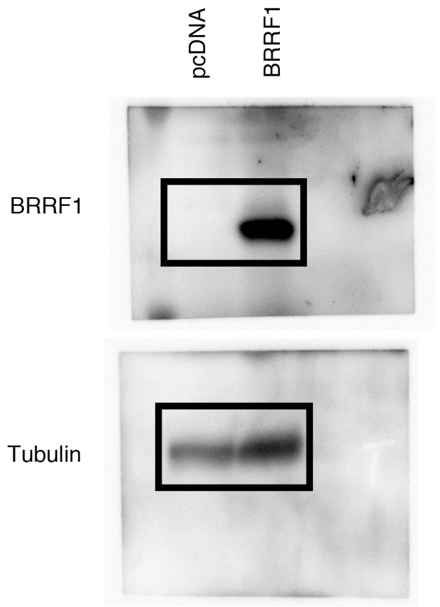

Images surrounded by ☐ were used for Fig. 6c

Supplementary Figure 18

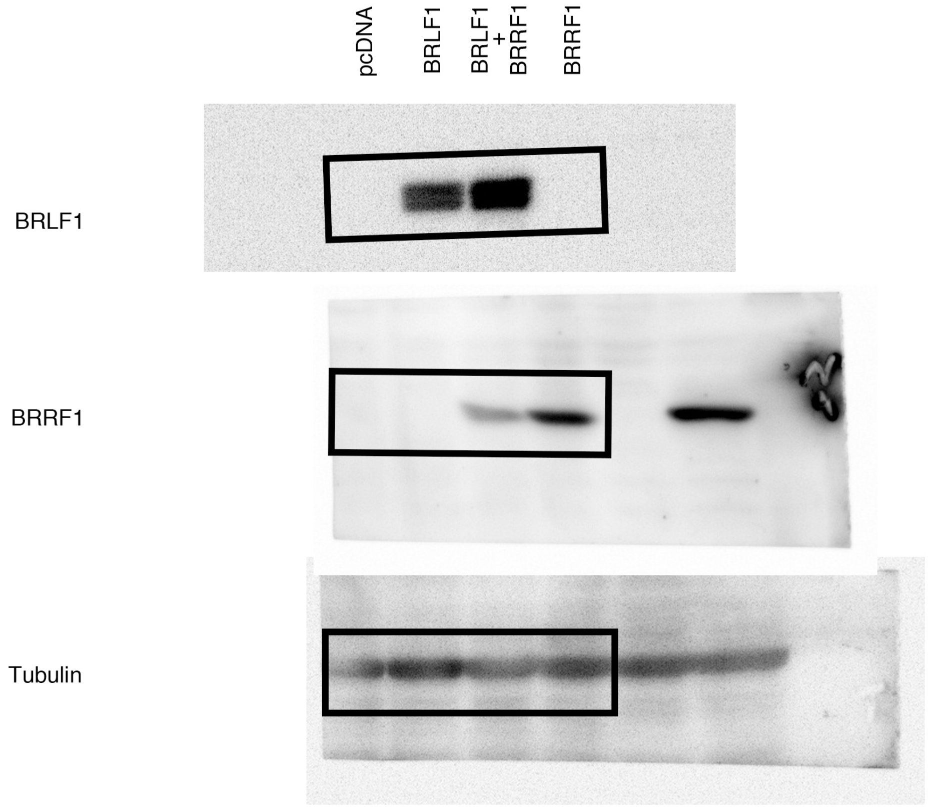

Images surrounded by ☐ were used for Fig. 6d

Supplementary Figure 19

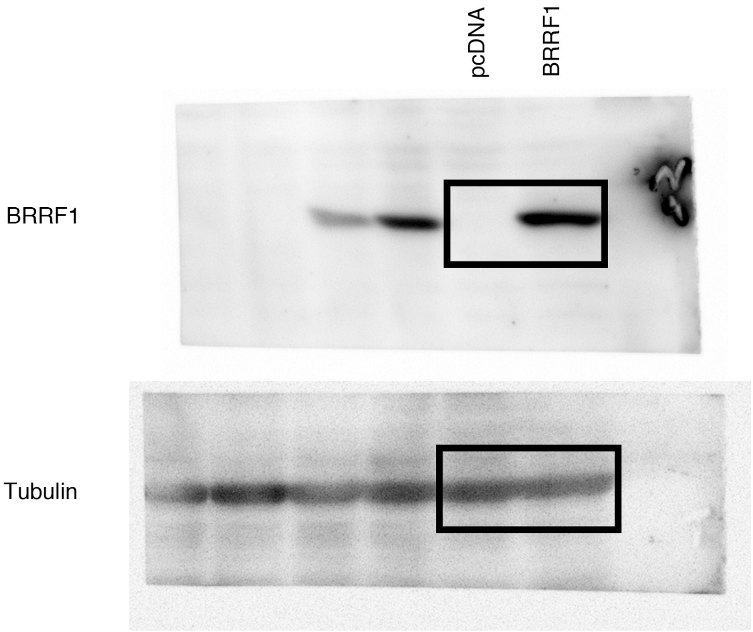

Images surrounded by ☐ were used for Fig. 6f

Supplementary Figure 20

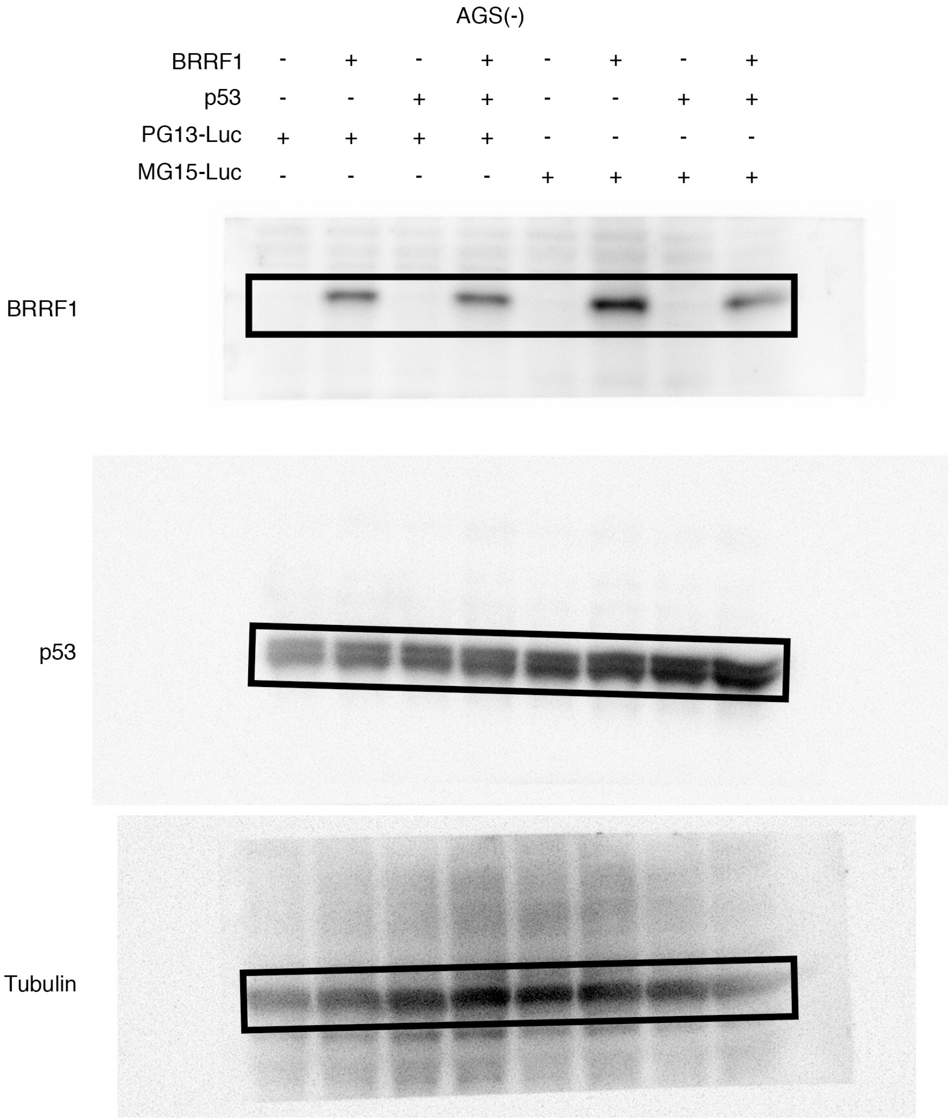

Images surrounded by □ were used for Fig. 7a

Supplementary Figure 21

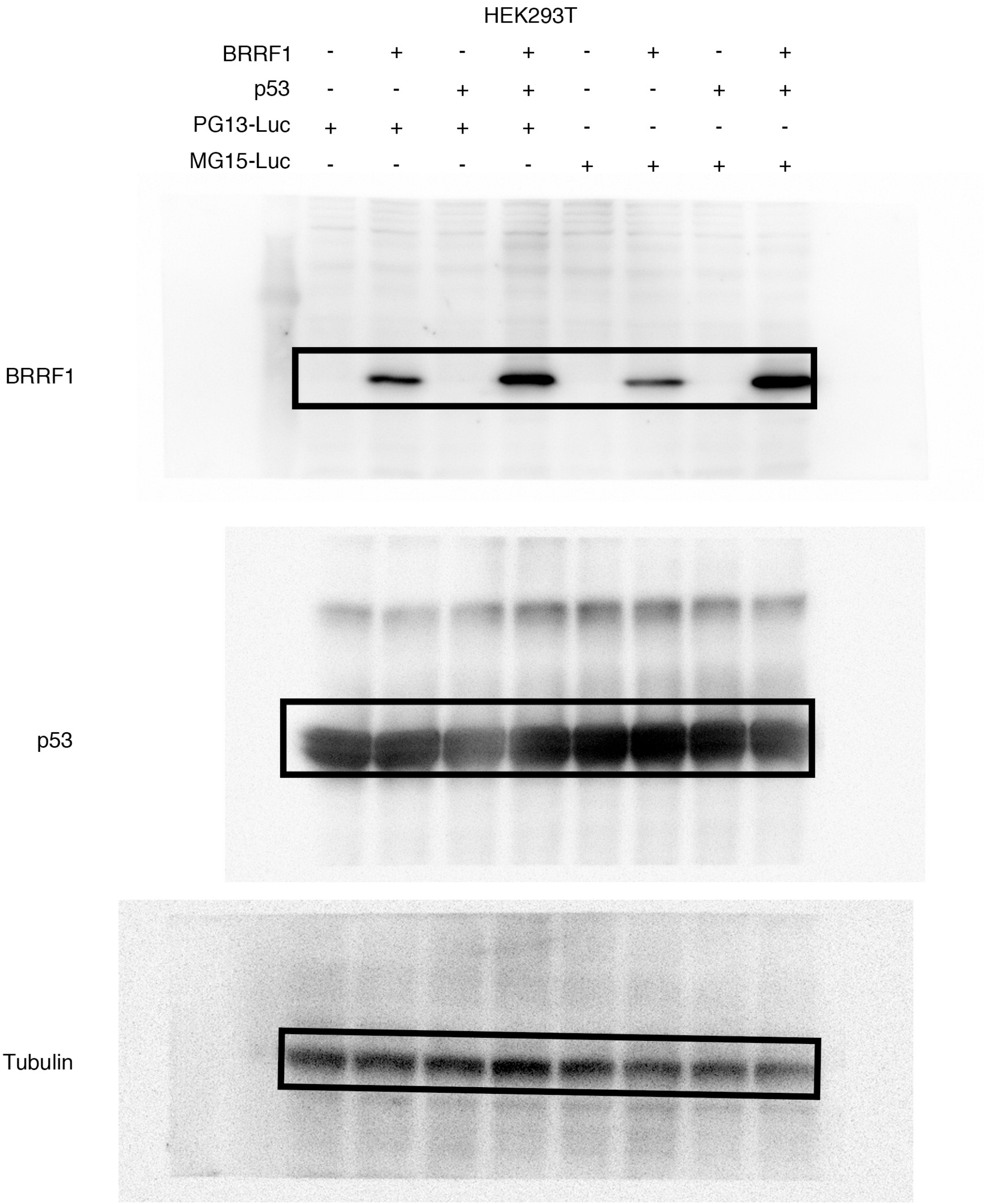

Images surrounded by ☐ were used for Fig. 7b

Supplementary Figure 22

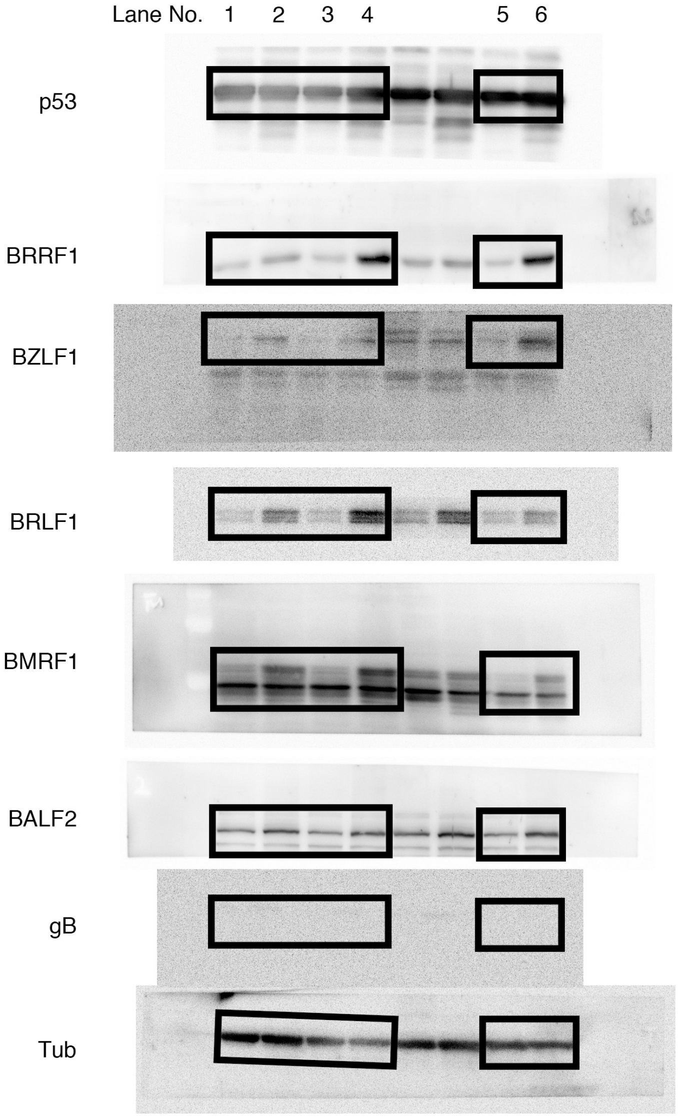

Images surrounded by □ were used for Fig. 8

Supplementary Figure 23

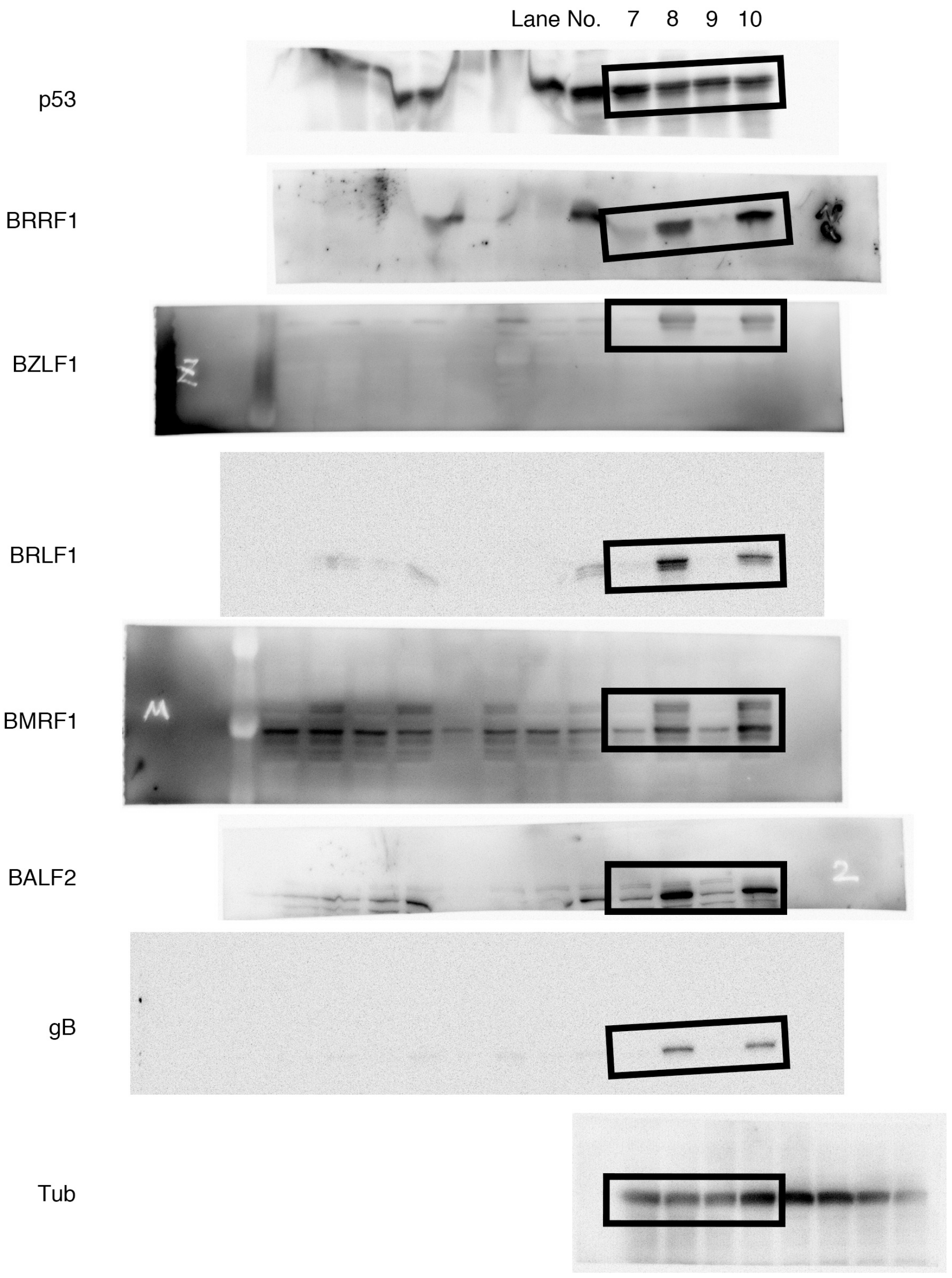

Images surrounded by ☐ were used for Fig. 8

Supplementary Figure 24

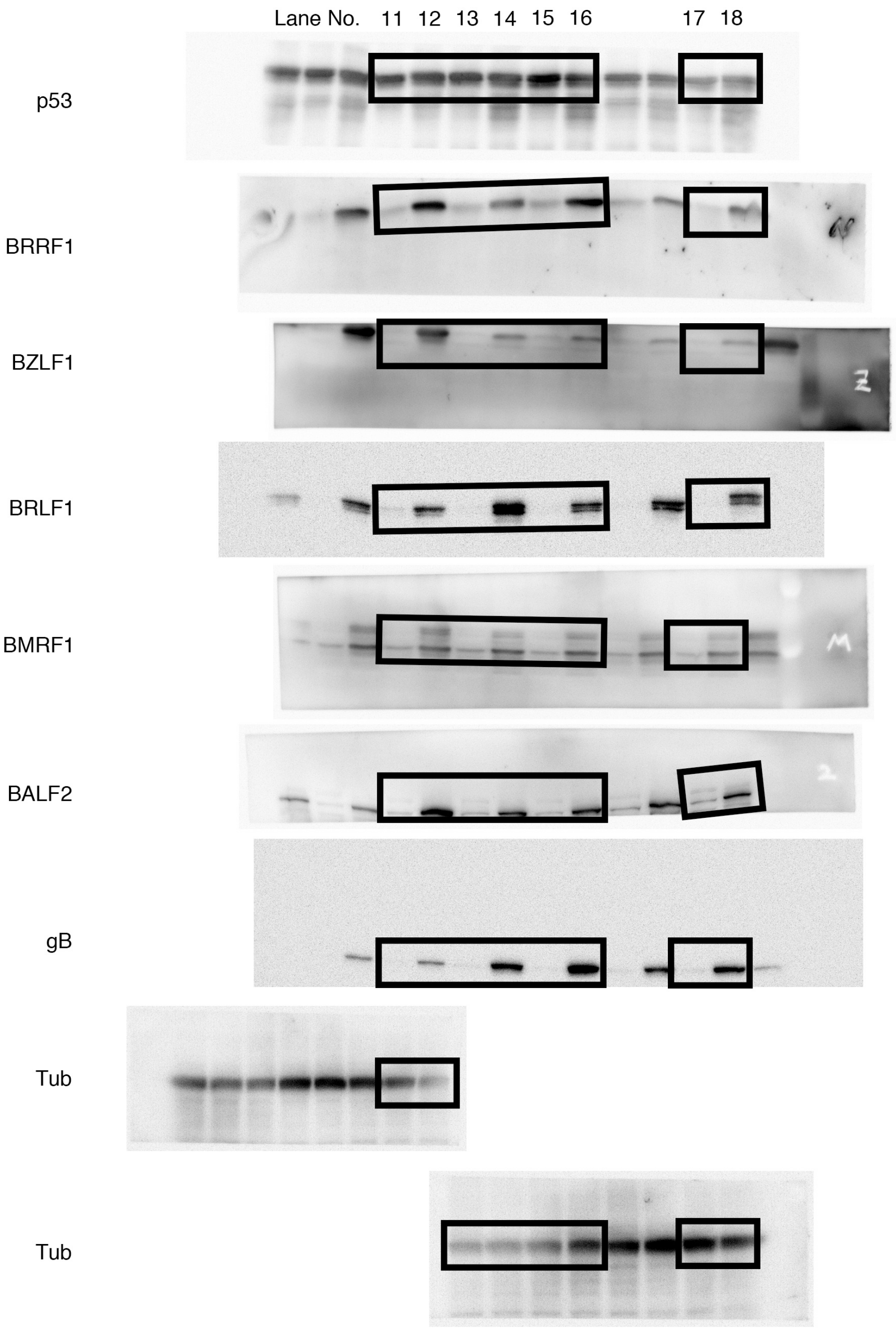

Images surrounded by ☐ were used for Fig. 8
